# Optimization of Female Head–Neck Model with Active Reflexive Cervical Muscles in Low Severity Rear Impact Collisions

**DOI:** 10.1007/s10439-020-02512-1

**Published:** 2020-04-24

**Authors:** I Putu A. Putra, Johan Iraeus, Fusako Sato, Mats Y. Svensson, Astrid Linder, Robert Thomson

**Affiliations:** 1 Department of Mechanics and Maritime Sciences, Chalmers University of Technology (Campus Lindholmen), Hörselgången 4, 41296 Gothenburg, Sweden; 2 Swedish National Road and Transport Institute (VTI), Regnbågsgatan 1, 41755 Gothenburg, Sweden; 3 Japan Automobile Research Institute, Tsukuba, Ibaraki Japan

**Keywords:** Finite element, Neck muscle reflex, Rear impact, Whiplash, Human body model

## Abstract

ViVA Open Human Body Model (HBM) is an open-source human body model that was developed to fill the gap of currently available models that lacked the average female size. In this study, the head–neck model of ViVA OpenHBM was further developed by adding active muscle controllers for the cervical muscles to represent the human neck muscle reflex system as studies have shown that cervical muscles influence head–neck kinematics during impacts. The muscle controller was calibrated by conducting optimization-based parameter identification of published-volunteer data. The effects of different calibration objectives to head–neck kinematics were analyzed and compared. In general, a model with active neck muscles improved the head–neck kinematics agreement with volunteer responses. The current study highlights the importance of including active muscle response to mimic the volunteer’s kinematics. A simple PD controller has found to be able to represent the behavior of the neck muscle reflex system. The optimum gains that defined the muscle controllers in the present study were able to be identified using optimizations. The present study provides a basis for describing an active muscle controller that can be used in future studies to investigate whiplash injuries in rear impacts

**Electronic supplementary material:**

The online version of this article (10.1007/s10439-020-02512-1) contains supplementary material, which is available to authorized users.

## Introduction

Finite element (FE) models of the human neck have been used to study cervical kinematics and injury response related to vehicle collisions since the early 1990s.^2^ These models are valuable tools for understanding cervical spine kinematics as well as injuries that result from vehicle collisions. Several FE models of the human neck have been developed^2^ and were aimed mostly to understand occupant kinematics during the crash as well as to predict tissue level injuries.

To use a head–neck FE model for assessing the risk of soft tissue neck injuries, so called whiplash injuries, the correct prediction of cervical spine kinematics is important. During rear impacts, several phases of neck kinematics are observed: retraction, extension and flexion.^10^,^11^,^39^ Many hypothesis regarding the causation of whiplash injuries are related to the retraction motion of the neck (S-shape cervical spine).^6^,^19^,^20^,^39^,^43^ Therefore, if a head–neck FE model is to be used for predicting whiplash injuries, an important aspect is that the model generates a human-like retraction motion.

To increase the kinematic validity and correctly capture human responses during impacts, inclusion of active muscles in the neck is important.^2^ In the neck, muscles are a major part of the neck structure in terms of volume and could affect injury risk of other anatomical sites in the neck.^34^ Cervical muscles activity have also been shown to change the head kinematics during impact.^15^,^32^–^34^

Several methods of modelling active cervical muscles have been proposed, with two major activation approaches called Open-loop and Closed-loop.^24^ In the Open-loop scheme, cervical muscle activations are pre-defined by previous simulations, experimental or optimization results. Therefore, a model with an open-loop muscle scheme can only be used for a specific application since the model is dependent on a defined activation input.^24^ Conversely, in the closed-loop scheme which is most often implemented using a Proportional Integral Derivative (PID) controller, the muscle activation is controlled by real-time response of a model which imitates the human’s feedback mechanism.^24^

Human feedback and reflex mechanisms are complicated systems that are not yet well established^1^,^3^,^4^,^9^ and consist of multisensory inputs that have different functions. However, the vestibular system and the muscle spindle have been historically highlighted to maintain the head orientation in space and head on trunk orientation by activating the cervical muscles.^1^,^3^,^4^,^9^ The vestibular system could initiate the Vestibulocollic reflex (VCR) by sensing the head rotational and translational motion. Meanwhile, the muscle spindle senses the changes in muscle length and could trigger the Cervicocolic reflex (CCR). 1,3,4,9

Several neuromuscular models have accounted for reflex responses initiated by the human neck muscle reflex system into their model of cervical muscles.^7^,^8^,^16^,^18^,^22^,^23^ However, none of these models were specifically developed to assess whiplash injury. Moreover, none of these models were developed to represent the 50th percentile female.

To address the lack of female representation in human body models, a human body model which represents the 50^th^ percentile female called ViVA OpenHBM was developed at the Chalmers University of Technology in Sweden.^25^,^26^ This model has been validated against PHMS (Post Mortem Human Subjects) data but not against volunteer data. To allow further developments, the ViVA OpenHBM was structured as a modular model and consisted of a whole-body model and a head–neck model.^26^ The head–neck model was also further developed by adding active reflexive cervical muscle controllers.^27^

In the latest study, Putra *et al*.^27^ implementing two different muscle controllers to the cervical muscles of the ViVA OpenHBM head–neck. One controller was implemented to approximate reflex response using a neck link orientation, and another controller was implemented to represent displacement feedback from the muscle spindle. The comparison of head kinematics between the models showed the improvement of the head kinematics agreement between the model and the volunteer responses. Better kinematics agreement was achieved by the model with an active muscle controller based on the neck link angle. Therefore, in this study, the muscle controller based on the neck link angle was further studied with three different approaches to identify the controller parameters.

The first objective of this study was to represent the cervical muscle reflex response with a closed-loop PD feedback control mechanism to the Finite Element (FE) models of cervical muscles. The second objective was to calibrate the PD control gains by an optimization-based parameter identification based on published-volunteer data and to analyze the effects of three calibration objectives to the head and cervical kinematics of the model.

## Material and Methods

### ViVA Open Human Body Model (ViVA OpenHBM) Head–Neck Model

The dimensions of the ViVA OpenHBM head–neck model^25^ correspond to the 50th percentile female stature as mentioned in Schneider *et al*.^31^ In the original version, the 34 cervical muscles are represented by 129 beam elements on each side with Hill-type material models implemented without any activation forces. The validation of the head–neck model in rear impacts was conducted against five female PHMS head–neck complexes as published by Stemper *et al*.^36^–^38^

The base for the current study is the simplified cervical spine ViVA OpenHBM head–neck model.^26^ This model was developed by removing the intervertebral non-muscular soft tissue structures and introducing compliant joints (translational, axial rotation, lateral bending and flexion-extension compliance) based on *(in vitro)* kinematic data of human subjects.^26^ These simplifications have been proven to save overall computational cost by about 39% with no significant difference in CORA ratings of the head–neck kinematics in rear-impact collisions.^26^

### Active Cervical Muscle Modelling

An active muscle model was developed to represent the neck muscle reflex system. In the present study, this controller is referred as the Angular-Positioned Feedback (APF) controller.

The cervical muscles in the present model were modelled using beam (resultant truss) elements with LS Dyna *MAT_156/*MAT_MUSCLE.^13^ The total force of each element is described in Eq. (1).

1 $$F = {\text{PCSA}} \cdot \sigma_{\hbox{max} } \left[ {N_{\text{a}} \left( t \right) \cdot f_{\text{v}} \left( v \right) \cdot f_{\text{l}} \left( l \right) + f_{\text{pe}} \left( l \right)} \right]$$

PCSA is the Physiological Cross-Sectional Area of each fascicle, $\sigma_{ \hbox{max} }$ is the maximum isometric stress, $N_{\text{a}} \left( t \right)$ is the muscle activation level with range of 0-1, and $f_{\text{v}} \left( v \right),\, f_{\text{l}} \left( l \right)$ $f_{\text{pe}} \left( l \right)$ are the force-velocity function, force-length functions, and force contribution from parallel elastic stiffness which were based on Winters and Stark.^40^ The detailed explanation regarding muscle modelling of the current model can be found in Östh *et al*.^25^

**Figure. 1:**
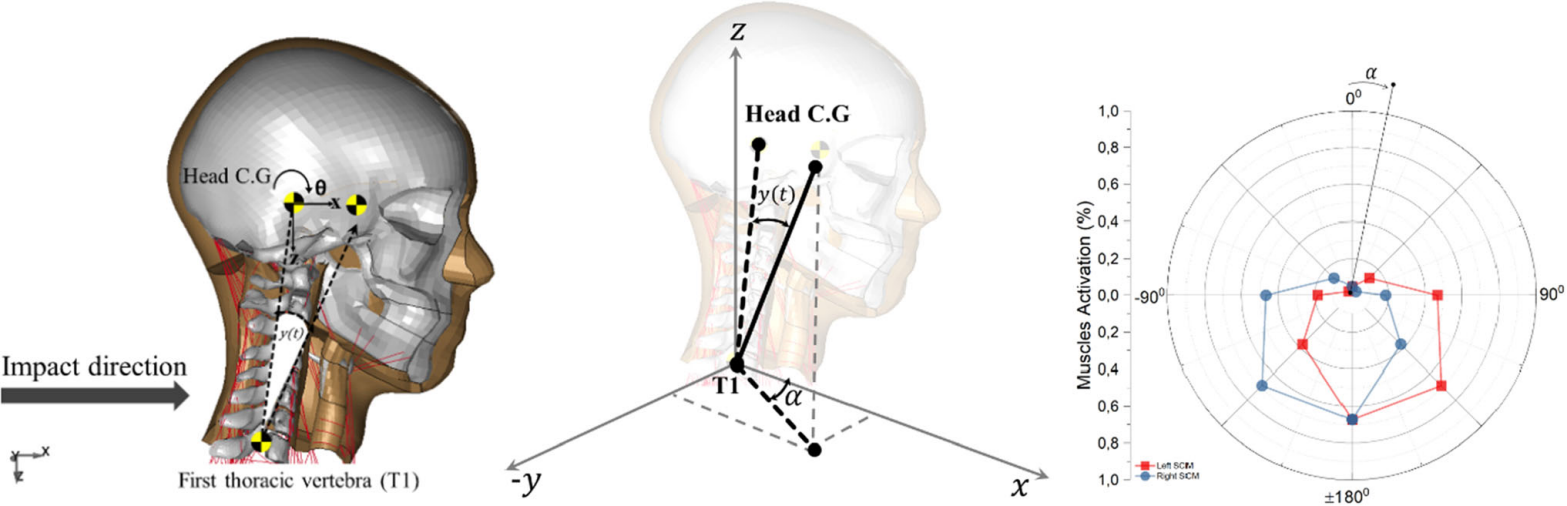
Simulation setup, controller vector calculation and projection using spatial tuning pattern.

The Proportional and Derivative (PD) controller defined by the PIDCTL function in LS-DYNA was used to represent the human neck muscle reflex system in order to give activation signal to the cervical muscles with purpose to keep head’s horizontal orientation during rear-impact. This approach was adopted from earlier studies that were conducted by Östh *et al*.^22^,^23^ and Olafssdotir *et al*.^18^ This controller design was used to determine the different neck muscle recruitments shown in the spatial tuning pattern developed in Olafssdotir *et al*.^17^ (Fig. 1). In the present study, the level of muscle activation of each muscle was controlled by the signal that comes from the PD controller where a minimum activation level (5%) was equivalent to co-contraction and assumed similar to the activation level of relax condition. The co-contraction is required to balance the head in the upright posture.

To approximate the reflex system, the coordinate of the head’s center of gravity (head C.G) and the center of first thoracic spine (T1) vertebral body were sampled (Fig. 1). These coordinates were defined as the sample coordinates and were used to define the controller vector.

When impact loads are applied to the model, the head C.G and T1 positions are sampled and used to update the controller vector. If there are any differences between the reference vector and the current vector, an error angle is calculated between these two vectors. The controller vector is also the input needed for the spatial tuning pattern.

**Figure. 2:**
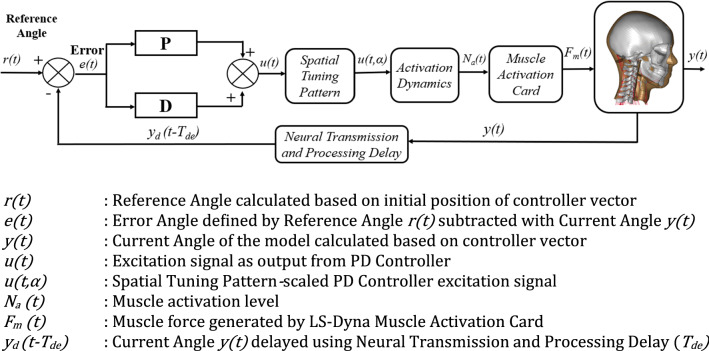
PD controller algorithm.

A delay was introduced in the error angle feedback signal in the feedback loop presented in Fig. 2 mimicking the neural processing delay. The delayed error signal was given to the PD controller, comparing this signal to the reference angle and then computing the control signal. The control signal was then given to a pre-defined spatial tuning pattern^17^ to define the specific muscle activation values for each muscle.

The muscle activation signals are filtered by the muscle activation dynamics function. The filtered signals then are input to the min-max function that can limit the lowest activation value to the value of co-contraction level and the maximum equal to 1.0. The final signal then goes to LS-DYNA Hill’s muscle activation card.

The muscle activation dynamics in the current study were modelled using two first order differential equation based on Winters and Stark^40^ as can be seen from the Eqs. (2) and (3).

2 $$\frac{{d{\text{Ne}}}}{dt} = \frac{{\left( {u - {\text{Ne}}} \right)}}{\text{Tne}}$$

3 $$\frac{{d{\text{Na}}}}{dt} = \left\{ {\begin{array}{*{20}c} {\frac{{\left( {{\text{Ne}} - {\text{Na}}} \right)}}{{{\text{Tna}},{\text{a}}}}, \,{\text{Ne}} \ge {\text{Na}}} \\ {\frac{{\left( {{\text{Ne}} - {\text{Na}}} \right)}}{{{\text{Tna}},{\text{d}}}}, \,{\text{Ne}} < {\text{Na}}} \\ \end{array} } \right.$$

where *u* is the muscle excitation signal, Ne is the neural excitation level, Na is the muscle activation level, Tna,a represents the muscle activation time, Tna,d is the muscle deactivation time, and Tne is the neural excitation time.

### Volunteer Data and Boundary Condition

Volunteer data from Sato *et al*.^29^ were used to calibrate the response of the model. The data are based on earlier experiments conducted by Ono *et al*.^20^ of low-speed rear impacts of two female volunteers. In the present study, the average of the first thoracic spine (T1) kinematics, both linear (*x*- and *z*-) and rotational (*y*-) displacements, were prescribed for the T1 of the model to replicate the kinematics of the volunteers. In addition, the sled displacement in *x*- direction was also added to the T1 of the model. To correctly mimic female cervical spine alignment, an average cervical spine alignment based on five female subjects from Sato *et al*.^30^ was adopted. A comparison of head kinematics in the passive model was also conducted by comparing two different cervical spine alignments, the original cervical spine alignment of the ViVA OpenHBM model^25^ and the present study cervical spine alignment which was based on Sato *et al*.^30^

### Optimization-Based Parameter Identification

The optimizations in this study were conducted using software called LS-OPT.^35^ The total length of each simulation was 400 with 100 ms gravitational acceleration (9.81 m/s^2^) settling included. These optimizations were aimed to identify the optimum PD gain values for the muscle controllers (Kp and Kd), the optimum neural transmission and processing time delay (Tnd), and the optimum time constants describing the muscle activation dynamics (Tna,a, Tna,d, and Tne). In total, there were six design variables or parameters in the present optimizations (Table 1).

**Table 1:** Optimization parameters.

Parameter/design variables	Symbol	Unit	Initial value	Optimization range
Proportional gain	Kp	%contraction/rad	0.601^a^	0.601^a^–40^b^
Derivative gain	Kd	%contraction/rad ms^−1^	412.62^a^	5^b^–412.62^a^
Neural transmission and processing delay	Tnd	ms	15^a^	3.5^c^–20^d^
Muscle activation dynamics				
Muscle activation time	Tna,a	ms	10^b,d,e^	5–15^f^
Muscle deactivation time	Tna,d	ms	40^b,d,e^	20–60^f^
Neural excitation time	Tne	ms	35^b,d,e^	20–50^f^

^a^Putra *et al*.^27^

^b^Östh *et al*.^22^

^c^Rosengren and Colebatch.^28^

^d^Ölafsdottir *et al*.^18^

^e^Winter and Stark.^40^

^f^Winter and Stark.^41^

The initial value and the lower bound of optimization range for the Kp was based on Putra *et al*.,^27^ while the upper bound was adopted from Östh *et al*.^22^ For the Kd, the initial value and the upper bound of the ranges were also based on Putra *et al*.,^27^ and the lower bound was based on Östh *et al*.^22^. The neural transmission delay value was set at 15 ms as the initial value,^27^ 3, 5 ms as the lower bound value^28^ and 20 ms as the upper bound value.^18^ For the time constants of the muscle activation dynamics, the initial values were based on original work from Winter and Stark,^40^ which were also adopted and implemented to the muscle controller by Östh *et al*.^22^ and Olafsdottir *et al*.^18^ Optimization ranges of time constants were adopted from Winter and Stark.^41^

The objective function of the optimizations was defined to minimize the error between model and volunteer data kinematics, which were used as the target. To calculate this error, a curve mapping algorithm based on Witowski *et al*.^42^ was used. The curve normalization was included in the algorithm to make sure that this method is independent of the measurement units. Four optimizations were conducted with three different strategies that minimized error between model and volunteer: linear and angular head kinematics, cervical spine angular kinematics, and head linear and angular direction and cervical spine angular kinematics (Table 2). Optimization 4 had similar objectives with Optimization 3 but the total weight of cervical spine objectives was equal to one.

**Table 2:** Optimization objectives.

Optimization name	Optimizations objectives function	Weight	Curve matching metric
Optimization 1 (Opt. 1)	To match volunteer head C.G *x*-disp	1	Curve mapping algorithm
	To match volunteer head C.G *z*-disp	1	
	To match volunteer head C.G rotational *y*-disp	1	
Optimization 2 (Opt. 2)	To match volunteer C1 absolute rotational *y*-disp	1	Curve mapping algorithm
	To match volunteer C2 absolute rotational *y*-disp	1	
	To match volunteer C3 absolute rotational *y*-disp	1	
	To match volunteer C4 absolute rotational *y*-disp	1	
	To match volunteer C5 absolute rotational *y*-disp	1	
	To match volunteer C6 absolute rotational *y*-disp	1	
	To match volunteer C7 absolute rotational *y*-disp	1	
Optimization 3 (Opt. 3)	To match volunteer head C.G *x*-disp	1	Curve mapping algorithm
	To match volunteer head C.G *z*-disp	1	
	To match volunteer head C.G rotational *y*-disp	1	
	To match volunteer C1 absolute rotational *y*-disp	1	
	To match volunteer C2 absolute rotational *y*-disp	1	
	To match volunteer C3 absolute rotational *y*-disp	1	
	To match volunteer C4 absolute rotational *y*-disp	1	
	To match volunteer C5 absolute rotational *y*-disp	1	
	To match volunteer C6 absolute rotational *y*-disp	1	
	To match volunteer C7 absolute rotational *y*-disp	1	
Optimization 4 (Opt. 4)	To match volunteer head C.G *x*-disp	1	Curve mapping algorithm
	To match volunteer head C.G *z*-disp	1	
	To match volunteer head C.G rotational *y*-disp	1	
	To match volunteer C1 absolute rotational *y*-disp	1/7	
	To match volunteer C2 absolute rotational *y*-disp	1/7	
	To match volunteer C3 absolute rotational *y*-disp	1/7	
	To match volunteer C4 absolute rotational *y*-disp	1/7	
	To match volunteer C5 absolute rotational *y*-disp	1/7	
	To match volunteer C6 absolute rotational *y*-disp	1/7	
	To match volunteer C7 absolute rotational *y*-disp	1/7	
Cross-Validation (Cross-Val.)	To match volunteer head C.G *x*-disp	1	Curve mapping algorithm
	To match volunteer head C.G *z*-disp	1	
	To match volunteer head C.G rotational *y*-disp	1	
Second-Validation (Opt.1 Val.)	To match volunteer head C.G *x*-disp	1	Curve mapping algorithm
	To match volunteer head C.G *z*-disp	1	
	To match volunteer head C.G rotational *y*-disp	1	

The method for optimization was the Metamodel-based Optimization using Sequential Response Surface Method (SRSM) with Domain Reduction.^35^ The Hybrid SA (Simulated Annealing + Leapfrog Optimizer for Constrained Minimization) was used as the optimization algorithm.^35^ For the metamodel, a Linear Polynomial Metamodel with D-Optimal point selection was used.^35^

The total number of Simulation Points (sub-iterations) was equal to 11 for each optimization iteration. The maximum number of global iterations was set to 10. The tolerance criteria were defined as ± 1% design change and ± 1% objective function tolerance as the LS-OPT default settings. See Stander *et al*.^35^ for detailed theories and explanations of the optimization strategy used in the present study.

### Cross-Validation Optimization

Cross-Validation with a different optimization strategy was conducted with a similar setup and objective as Optimization 1 (Tables 1 and 2) to verify the parameter identification. Instead of using a metamodel-based approach, direct optimization using The Genetic Algorithm was conducted.^35^ The total process was limited to 10 iterations with one verification iteration. Each iteration consisted of 30 simulations. The optimum parameters as the results of cross-validation optimization were then selected as the starting point of the parameters in a second validation optimization (Opt.1 Val) to identify if the result of optimum parameters was independent of the optimization strategy. The same optimization setup and strategy as the Opt.1 was selected as the Opt.1 Val. The optimization was conducted for Kp and Kd only while the other time parameters were kept constant.

### Quantitative Ratings Evaluation

To quantify the similarities between the kinematics responses of the model and the volunteers, an objective rating evaluation using Correlation Analysis (CORAplus) software 4.0.4 was conducted.^5^ Default corridors for CORA was selected with 5 and 50% of inner and outer limits respectively and combined with CORA correlation method to get the final score. The evaluation compared the whole duration of head kinematics between the model and volunteers’ average responses (0–300 ms). The comparison only considered the interval of 0–180 ms for the cervical spine displacement due to the data availability.

### Software and Computational Environment

The simulations were conducted using LS-Dyna^13^ ver9.2 double precision with LS-Prepost 4.5(64-bit)^14^ and OriginPro 2018(64-bit)^21^ were used as pre- and post- processing software.

## Results

### Optimization-Based Parameter Identification Results

#### Parameter Convergence

**Figure. 3:**
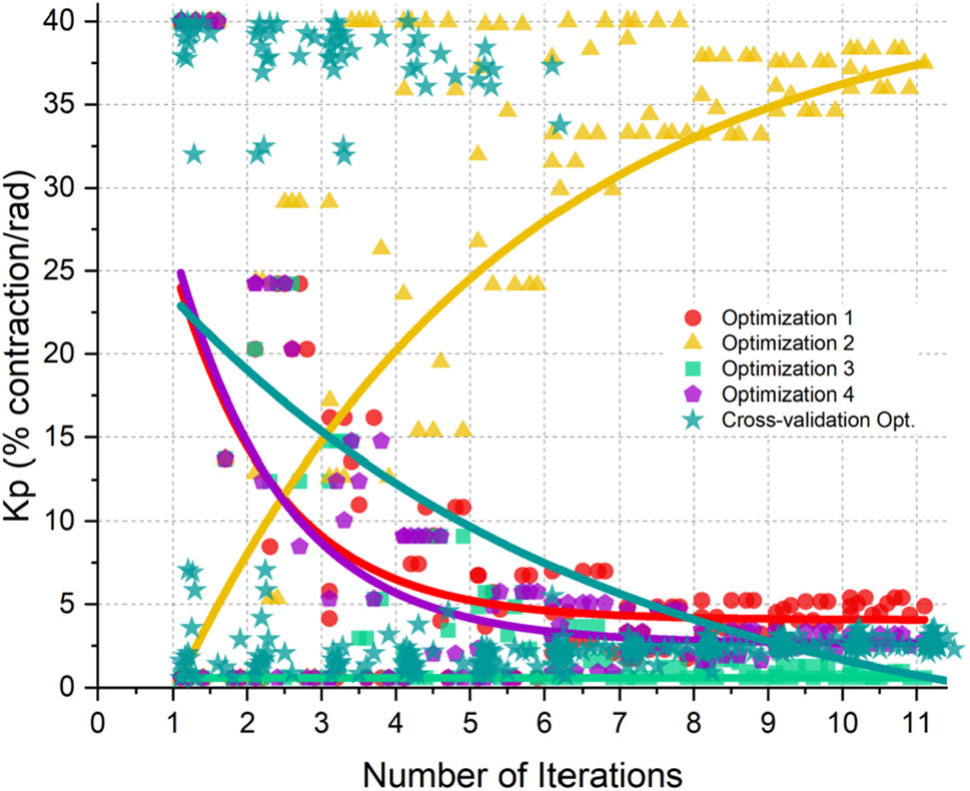
Convergence plots of Kp. Convergence plots for all parameters are attached in the Supplemental Material.

The example of a parameter convergence plot (Kp) is presented in Fig. 3. Convergence plots for all six parameters are attached in the Supplemental Material. The scatter plot represents the value of the simulated parameters and the thick line representing the trend, which was fitted using an exponential fit.^21^ All parameters were observed to convergence near the last iterations. All parameters converged to values within their assigned limits whereas Kd did converge to its lower limit for some optimization strategies.

#### Correlation Between Parameters

The linear correlation matrix between each parameter in all optimizations is presented in Table 3. A weak correlation (≤ ± 0.29) was seen in almost all optimizations. Five parameters had medium correlations (± 0.30 to ± 0.49) in Optimization 3, three parameters in Opt. 1, and Opt. 2, and two parameters in Opt 4. Only one strong correlation (± 0.50 to ± 1.00) was found between two parameters in Opt. 2.

**Table 3:**
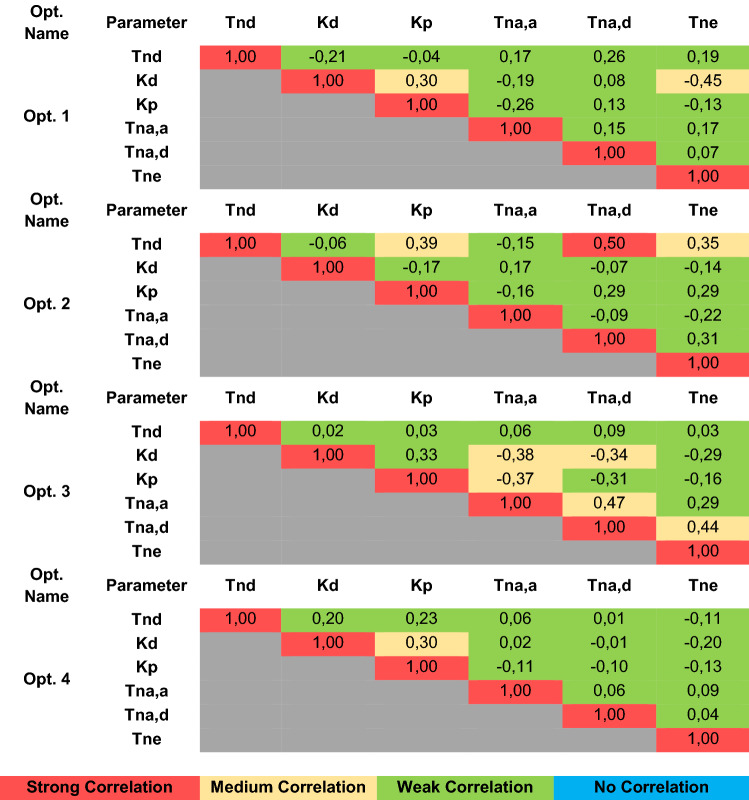
Linear correlation matrix between parameters.

#### Optimum Controller Parameters

The optimum controller parameters as the result of optimizations simulation are presented in Table 4. The optimum Proportional gain (Kp) for all simulations tended towards the minimum value except for Optimization 2, which instead moved towards the maximum limit. For the parameter Kd, the lower limit was the optimum value. However, different optimum values for Kd were obtained by the Opt. 2 and Cross-Validation Optimization. The optimum values for the time constants (Tnd, Tna,a, Tna,d, and Tne) describing neural delay and muscle activation dynamics were reasonably consistent and essentially independent of the optimization strategy.

**Table 4:** PID gains and optimum time delays.

Parameter	Unit						
		Opt. 1	Opt. 2	Opt. 3	Opt. 4	Cross-Val.	Opt. 1 Val.
Proportional gain (Kp)	%contraction/rad	4.89	37.49	0.60	2.85	3.08	6.02
Derivative gain (Kd)	%contraction/rad ms^−1^	5.00	106.34	5.00	5.00	85.16	5.00
Neural transmission and processing delay (Tnd)	ms	15.07	19.83	12.48	8.03	19.95	19.95
Muscle activation time (Tna,a)	ms	14.92	7.63	14.92	11.09	13.63	13.63
Muscle deactivation time (Tna,d)	ms	42.49	56.67	59.97	40.91	56.57	56.57
Neural excitation time (Tne)	ms	49.99	39.82	46.08	42.24	49.66	49.66

### Cross-Validation Optimization

A cross-validation optimization was conducted to identify the influence of the optimization method. All six parameters were observed to convergence and are reported in Table 4 (see Supplemental Material for details). When the optimum parameters were compared between the Opt. 1 and the Cross-Validation optimization, the differences were quite small except for the Kd (Table 4).

The second validation approach (as described in the methods section) utilized the output from the cross validation as the starting condition for a new optimization of strategy Opt. This simulation resulted in similar values to the original Opt.1 simulation (Table 4).

**Figure 4:**
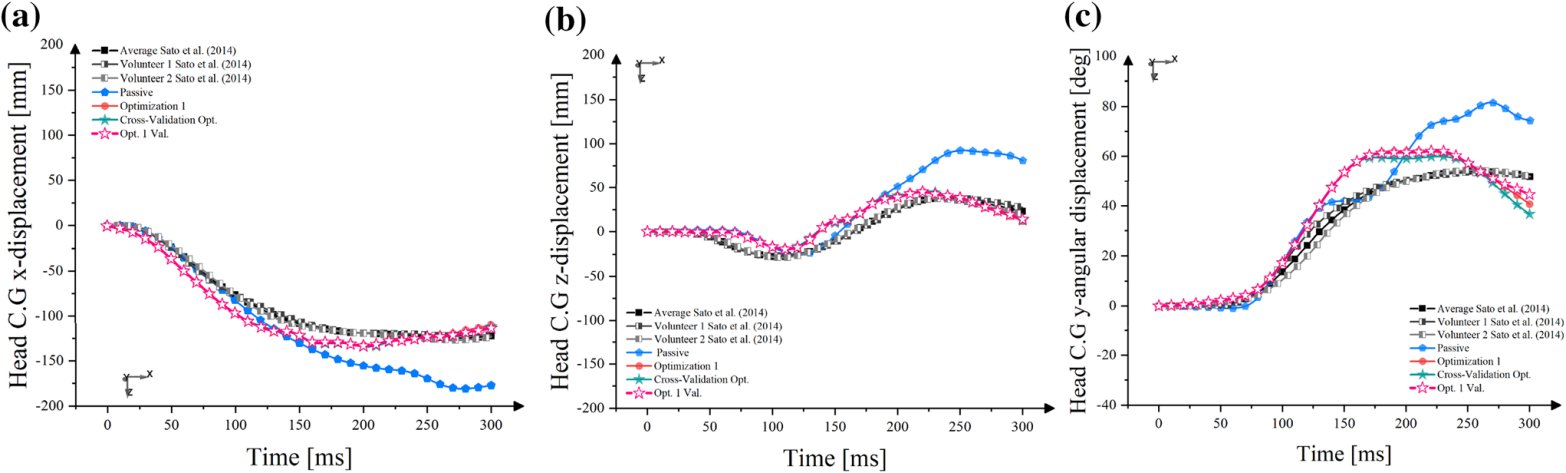
Head C.G. displacement comparison between optimization 1 and cross-validation optimization; (a) head C.G. *x*-displacement; (b) head C.G. *z*-displacement; (c) head C.G. *ry*-displacement.

The head kinematics results were also compared between the Opt. 1 model, the Cross-Val. model and the Opt.1 Val. model as shown in Figs. 4a–4c. In general, the head C.G. displacements were almost identical, with slightly better responses in the Opt.1 Val. model.

### Kinematics Comparison Between ViVA OpenHBM Head–Neck and Volunteer

#### Influence of Cervical Spine Alignment to Head Kinematics of Passive Models

**Figure 5:**
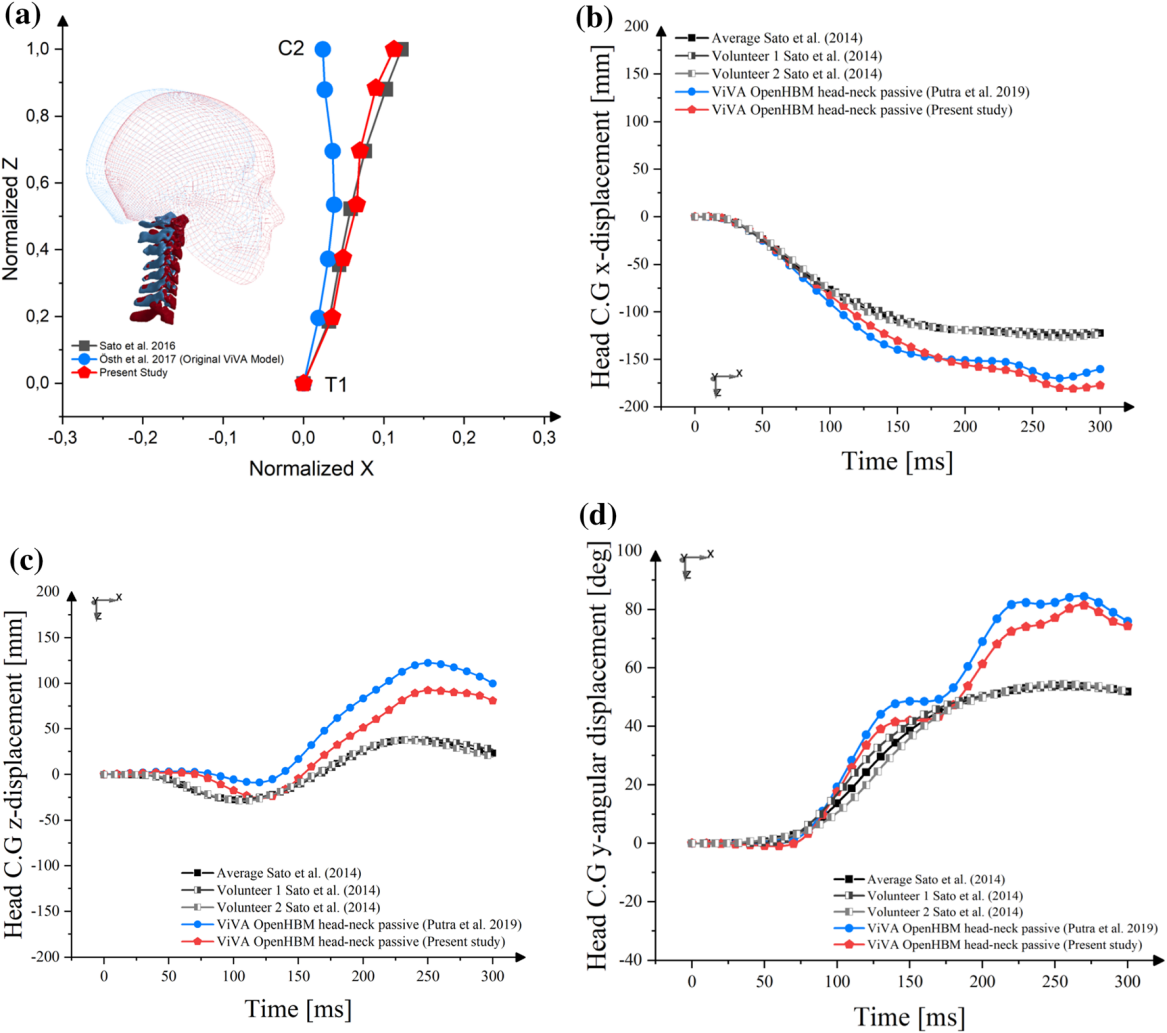
Influence of cervical spine alignment to head kinematics of passive model. (a) cervical spine alignment comparison; (b). head *x*-displacement; (c) head *z*-displacement; (d) head *ry*-displacement.

The average female cervical spine alignment based on Sato *et al*. (2016) was more kyphotic compared to the original ViVA OpenHBM head–neck model (Fig. 5a). In general, improved head kinematics agreement was seen when the model is configured to better reflect the average female cervical spine alignment. The difference in cervical spine alignment affected the head displacements in all directions, with a more pronounced difference observed in the *z*-displacement (Fig. 5c). With a new cervical spine alignment, the present study model could better reproduce the volunteer’s upward motion, which occurred at impact time around 100 ms.

#### Comparison of Head Kinematics

**Figure 6:**
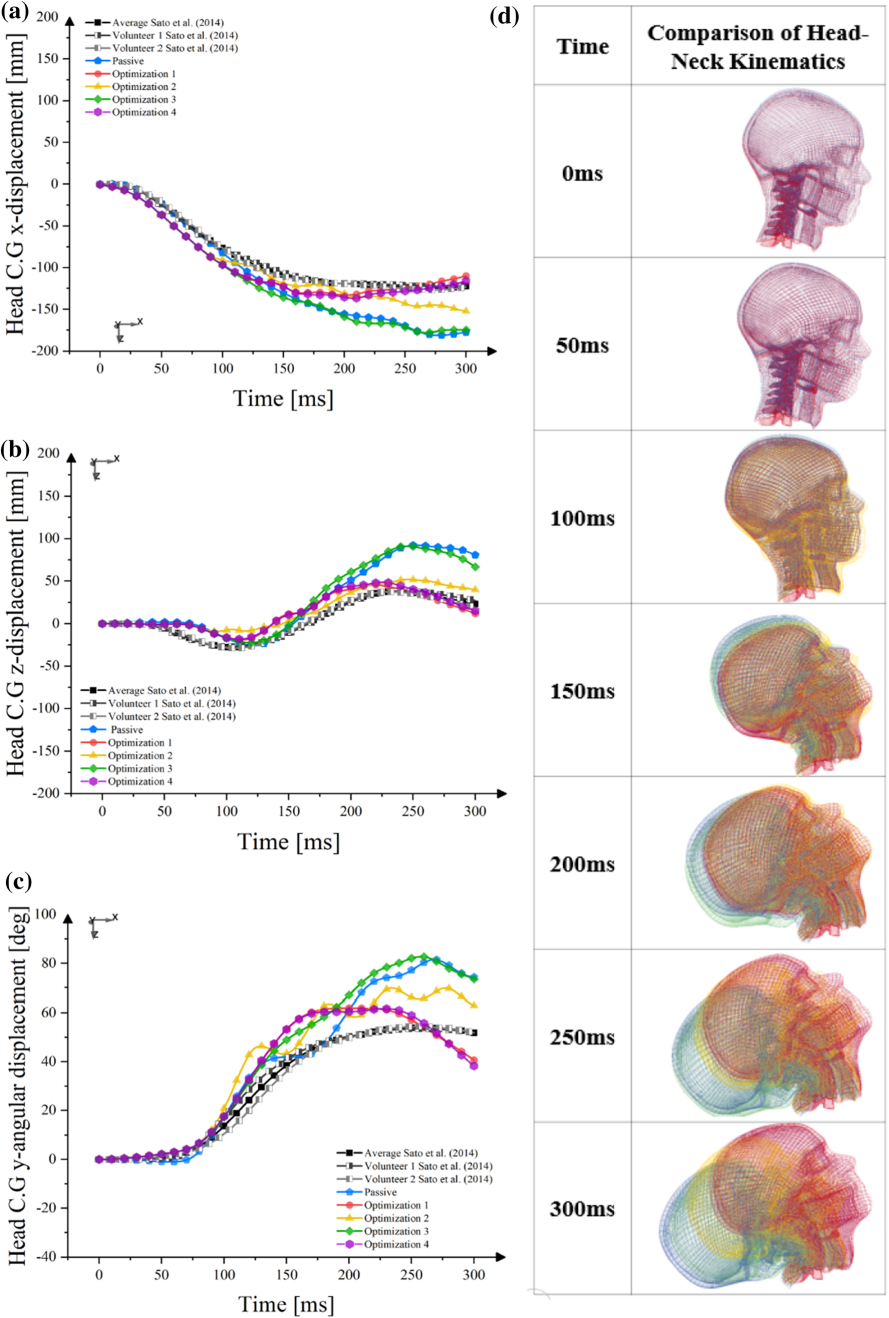
Comparison of head kinematics calibration simulation: (a) head C.G *x*-linear displacement; (b) head C.G *z*-linear displacement; (c) head C.G *y*-angular displacement; (d) time-series kinematics comparison.

The head kinematics comparison between the models with and without an active muscle controller can be seen in Fig. 6. In general, muscle activation alters the head kinematics by reducing peak displacements in all kinematics direction except for the model with optimum parameter from Optimization 3 (Fig. 6d).

Muscle activation started to change the head kinematics in the horizontal direction (*x*-displacements) at around 100 ms (Fig. 6a). The Opt. 1, Opt. 2, and Opt. 4 model closely followed the volunteer head kinematics until around 220 ms. After that, the Opt. 2 model started to deviate. The other two models were able to follow the volunteer head horizontal kinematics until 300 ms. The same head displacement was observed in the passive and Opt. 3 model for the whole duration of the simulation.

The comparison of vertical head motions (*z*-displacements) for the models with an active muscle controller, passive model, and the volunteer responses are presented in Fig. 6b. All models, including the passive model, could not follow the upward motion of the volunteer responses during the period of 50-100 ms. Three models could reduce excessive head vertical motions (Opt 1, Opt. 2 and Opt. 4). All three models closely followed the kinematic trend, although the displacement magnitude was not perfectly identical. A small difference was observed in the Opt. 3 model when compared to the model without an active muscle controller (Passive).

The head angular-y displacement is presented in Fig. 6c. All models over predicted this motion until impact time 125 ms. After that, the passive model started to perform within the range of volunteer responses. However, the models with active muscle controllers continued to exhibit rotational motions higher than the volunteer response until 150 ms. The Opt. 3 model started to deviate at 175 ms and had higher rotational movements after 175 ms when compared to the other models before following the passive model motion at impact time around 250 ms. Meanwhile, The Opt. 2 model could reduce the head rotational *y*-displacement after 175 ms. An almost identical rotational motion was observed in the Opt. 1 and Opt. 4 model.

#### Comparison of Cervical Spine Kinematics

**Figure 7:**
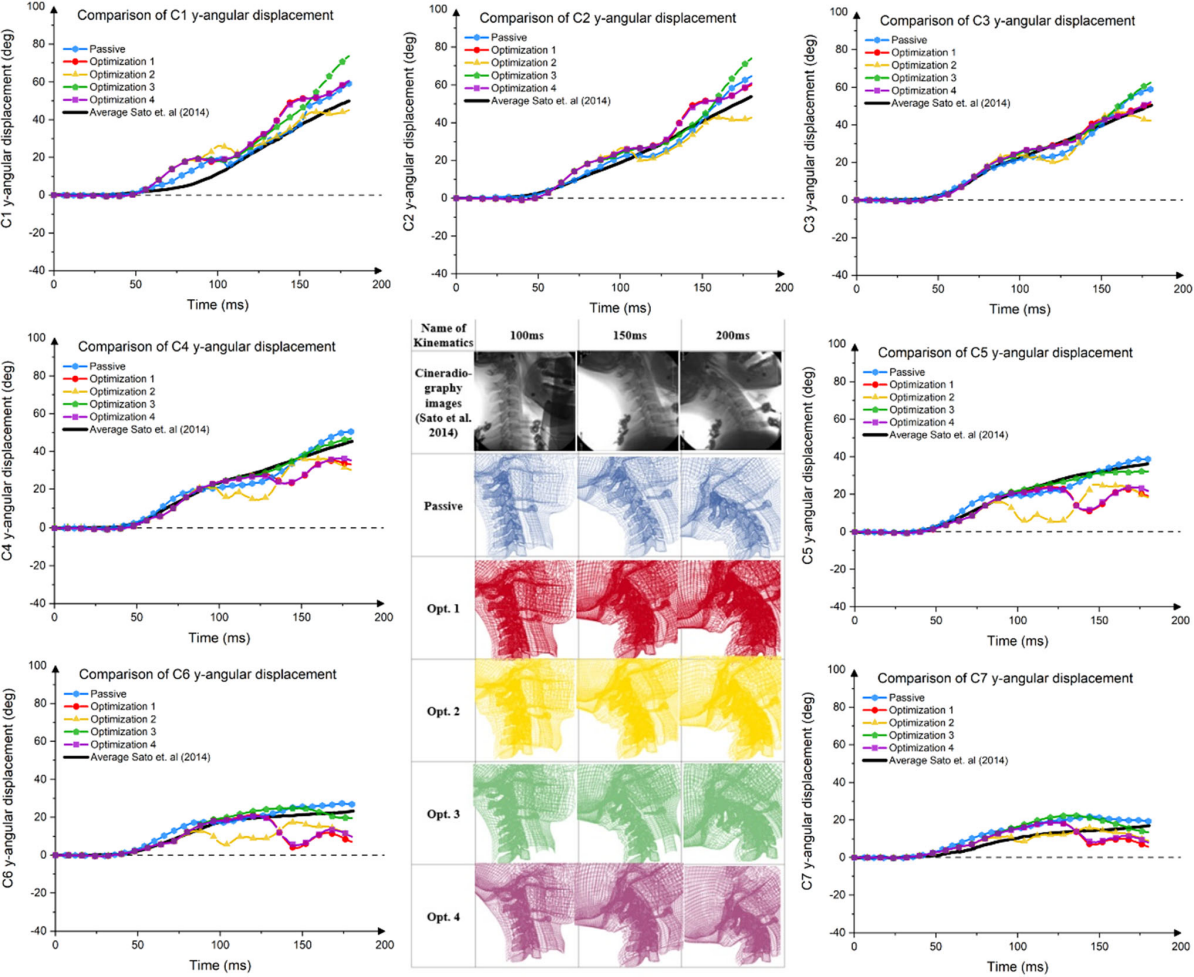
Comparison of cervical spine kinematics.

The comparisons of absolute *y*-angular displacement between the C1–C7 of the model and the average of volunteers are plotted in Fig. 7.

Both active and passive models over-predicted the C1 angular response of the volunteer, especially during the impact time of 50–100 ms. For the next 50 ms, the Passive and Opt. 2 model could follow the volunteer response but not in the other models.

The kinematics comparison between all models in C2 *y*-angular displacement showed that all models could not replicate the kinematics of volunteers until 100 ms except for the passive model. After that, all models deviated.

All models could reasonably produce the volunteer C3 *y*-angular displacement until 100 ms. After that, Opt. 1 and Opt. 4 model could match the volunteer’s response until 200 ms but only up to 150 ms for the Opt. 3 model. Deviation from the volunteer’s kinematic target was more pronounce in the passive and Opt. 2 model.

Until 120 ms, two models (Opt. 1 and Opt. 4) could reasonably mimic the volunteer kinematic responses when the kinematics of C4 *y*-angular were compared to the volunteer responses. The most substantial deviation was observed in the Opt. 2 model. A similar trend was also observed for the C5 and C6 *y*-angular displacement.

For the rotational motion of the C7, generally, all models could replicate the kinematics of the volunteers at least until 140 ms, although it was not perfectly identical. After that, two models (Opt. 1 and Opt. 4 model) started to deviate, while other models were following the volunteer displacement.

### Quantitative Rating Evaluation

The quantitative rating evaluation using CORA is presented in Table 5. The model that was calibrated for head kinematics only with initial value based on Cross-Validation (Opt.1 Val.) improved the head kinematics agreement the most compared to other models. This model also had the highest score for all head kinematics components. For the cervical spine kinematics, the highest average agreement was achieved by the Opt. 3 model, although it was almost identical with the passive model. When both kinematics components were combined, Cross-Val. model had the highest agreement with the volunteers’ responses, almost similar rating with the Opt.1 Val. model.

**Table 5:** Quantitative rating evaluation using CORA.

Kinematics parameter	Opt. 1	Opt. 2	Opt. 3	Opt. 4	Cross-Val.	Opt.1 Val	Passive
Head *x*-linear displacement	0.874	0.858	0.651	0.864	0.874	0.878*	0.715
Head *z*-linear displacement	0.577	0.483	0.463	0.562	0.582	0.593*	0.465
Head *y*-angular displacement	0.806	0.741	0.691	0.804	0.811	0.813*	0.742
Average of head displacement	0.752	0.694	0.602	0.743	0.755	0.761*	0.640
C1 *y*-angular displacement	0.774	0.847	0.768	0.771	0.778	0.780	0.896*
C2 *y*-angular displacement	0.892	0.896	0.883	0.892	0.899	0.898	0.936*
C3 *y*-angular displacement	0.986	0.949	0.949	0.988	0.989*	0.986	0.936
C4 *y*-angular displacement	0.880	0.836	0.995*	0.884	0.876	0.876	0.944
C5 *y*-angular displacement	0.750	0.647	0.980*	0.757	0.747	0.744	0.938
C6 *y*-angular displacement	0.718	0.682	0.896*	0.742	0.728	0.711	0.857
C7 *y*-angular displacement	0.645	0.870*	0.675	0.679	0.677	0.648	0.625
Average of cervical spine angular displacement	0.806	0.818	0.878*	0.816	0.813	0.806	0.877
Average of head and cervical spine displacement	0.779	0.756	0.740	0.780	0.784*	0.783	0.758

*Highest value

## Discussion

The 50th Percentile female HBM called ViVA OpenHBM^25^,^26^ was further developed in this study by implementing active muscle controllers to represent the human neck muscle reflex system.

The implementation of a PID controller to represent the human neck muscle reflex system in the current study was adopted from Östh *et al*.^22^,^23^ and Olafssdotir *et al*.^18^ Beside these studies, other studies implemented active muscle control representing feedback response from reflexes steered by the human neck muscle reflex system.^7^,^8^,^16^,^18^,^22^,^23^ These load cases were different from the current study as none of them evaluated the model against volunteer tests in a rear-impact collision. Evaluating the performance of such controllers is also relevant for rear impacts as most of the drivers or occupants do not notice that they will be impacted by another car from behind.

There are no confirmed injury mechanisms that predict whiplash systems. The current hypotheses under investigation^6^,^19^,^20^,^39^,^43^ focus on the formation of a cervical S-shape that tends to occur in before 150 ms after impact. Spine kinematics that can accurately predict the head–neck kinematics up to 150 ms are thus a priority.

The optimizations used for parameter identification converged for all six parameters of interest. Therefore, it was believed that the global minimum of each parameter, with relation to three different optimization objectives, had been found. When the independency of each parameter was analyzed, it was found that only Optimization 2 (when only neck kinematics used as the target of optimization) had two parameters with a strong correlation suggesting this approach was not worth pursuing. For the other optimization objectives, most of the parameters had only had weak correlations. These results indicate the selected control parameters are independent of each other.

The optimum parameters of the derivative gain, Kd, converged to a similar value for Optimization 1, 3, and 4. For proportional gain, Kp, some variations were observed in those same optimizations. This suggests that the parameter Kd was less sensitive to the objective function for the optimization, and the derivative control aspect of the present controller has less authority than the Kp on the neck kinematics.

The parameters of cross-validation simulation converged at almost identical values with the Opt.1. Only the value of the Kd was quite different. When the head C.G. kinematics of the two optimization techniques were directly compared, it was found that the cross-validation model had an almost identical response, although the optimizations strategy was different. This result again demonstrated that the parameters of Kd only had a small influence on the optimization objectives.

When the cervical spine alignment of the model was adjusted to the average female alignment,^30^ improvement of head kinematics (mainly in the vertical motion) was already achieved in the passive model. This result highlighted that the initial alignment of the model cervical spine is essential to be evaluated before implementing and optimizing the active muscle controller to the model.

When the head and cervical spine kinematics responses of the models and the volunteers were compared, muscle activation were shown to alter the head kinematics by reducing peak displacements. However, when the muscles were activated, the tension from the muscles caused cervical spine buckling, especially after impact time 150 ms. This was observed in Optimization 1 and Optimization 4. Less contraction of cervical muscles was observed in Optimization 3 and less cervical spine buckling was observed. Consequently, the head kinematics of Optimization 3 was almost identical with the passive model.

Even though only cervical spine kinematics were used as the objective in Optimization 2, the cervical spine kinematics agreement was still below that found in Optimization 3. This result also proved that the current simplification of neck muscle reflex system by using a vector between head C.G. and the center of the T1 vertebral body combined with spatial tuning pattern for activated the cervical muscle worked well to detect the head position changes. However, it had less influence to control the cervical spine kinematics. To better control the cervical spine kinematics, it could be beneficial to define an error signal that incorporates more degrees of freedom in the head–neck complex. Another approach would be to include a controller that mimics the displacement feedback from the neck muscle spindles. In fact, high concentrations of muscle spindle were found in the deep neck muscles,^12^ which connected directly with the cervical spine. This also may address the complex intravertebral kinematics and ensuring muscle activity is within biomechanical limits. This extra control implementation would require more parameter identification studies (similar to the APF controller described herein). Due to the complex muscle configurations, this additional controller design was beyond the scope of the current study.

The current controller implementation assumes the neck muscle reflex system controlling the muscle response can be captured by a single rigid body link between T1 and the head CG. As this assumption ignores individual rotations within the vertebral joints, the existing controller does not explicitly capture cervical kinematics and implicitly accounts for this behavior through the different optimization approaches (Opt. 2–4). The controller design could be updated to include two or three feedback signals better describing the head–neck kinematics. Increasing the control complexity would require establishing a new spatial tuning pattern to describe the muscle recruitment strategy. Increasing the controller complexity creates higher requirements on the type and quantity of validation data, and the current model currently uses the most of available volunteer data.

The current study highlights the importance of including active muscle response to mimic the volunteer’s kinematics. A simple PD controller has found to be able to represent the behavior of the neck muscle reflex system. The optimum gains that defined the muscle controllers in the present study were able to be identified using optimizations. The different optimization approaches could refine the model response incrementally but could not perfectly reproduce the volunteer response. The present study provides a basis for describing an active muscle controller that can be used in future studies to investigate whiplash injuries in rear impacts.

## Electronic supplementary material

Below is the link to the electronic supplementary material.

Supplementary material 1 (DOCX 3116 kb)
