# Supplementary material for: Optimization of Female Head–Neck Model with Active Reflexive Cervical Muscles in Low Severity Rear Impact Collisions
Source: Ann Biomed Eng. 2020 Apr 24;49(1):115–28. doi: 10.1007/s10439-020-02512-1 (PMC7773618; doi:10.1007/s10439-020-02512-1)

**SUPPLEMENTAL MATERIAL**

**Convergence Plot of Kp**


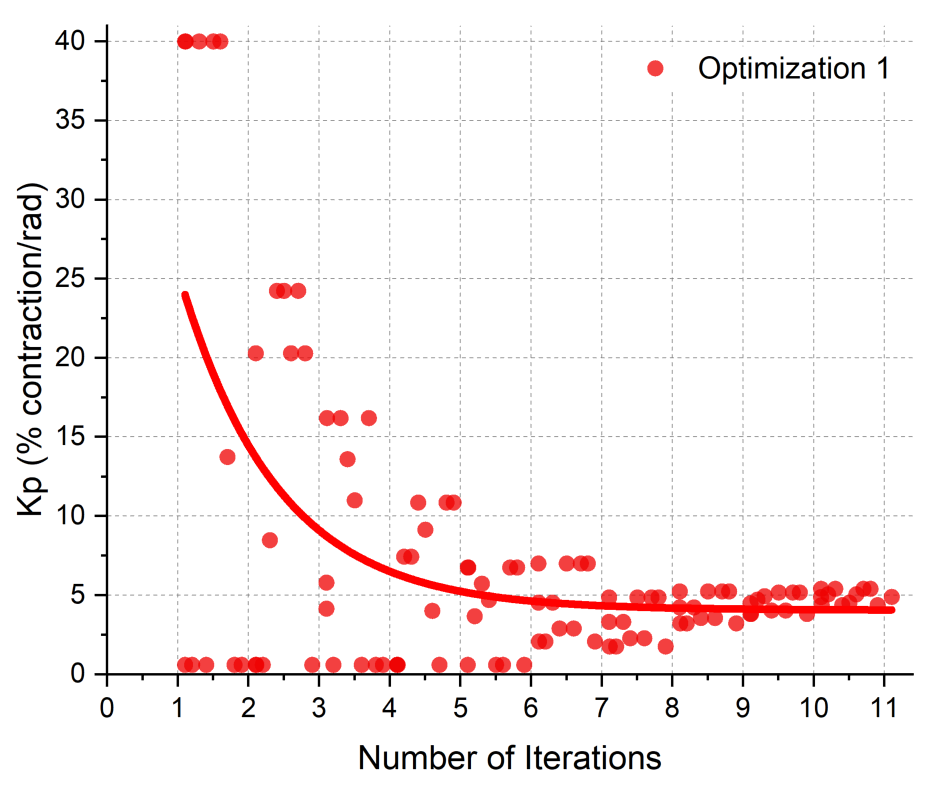

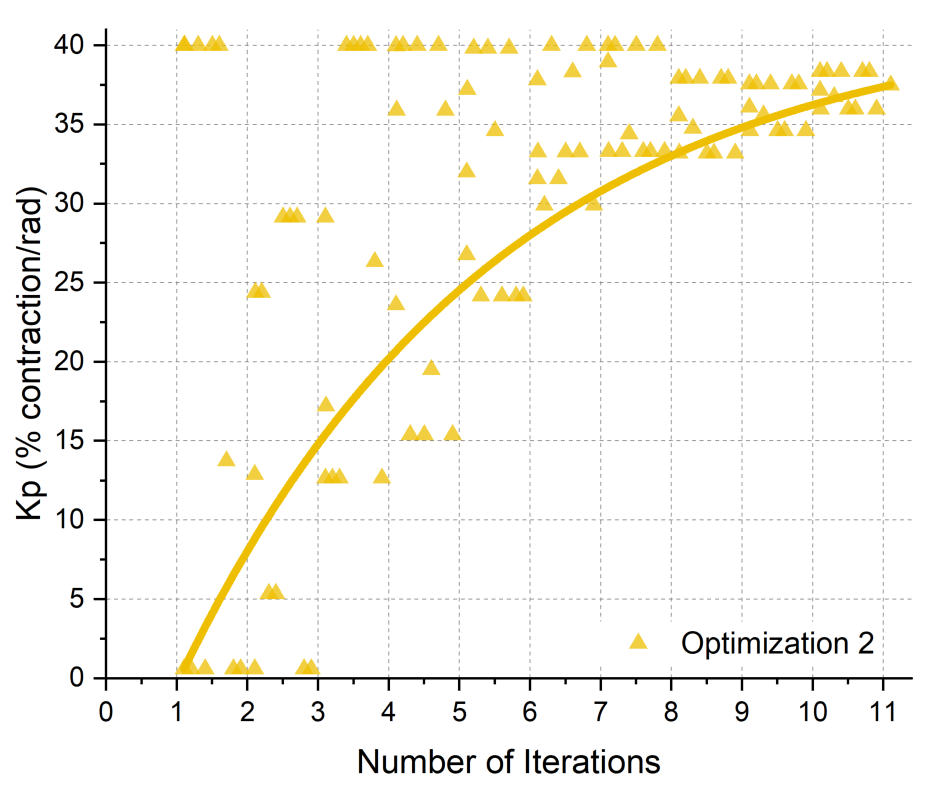


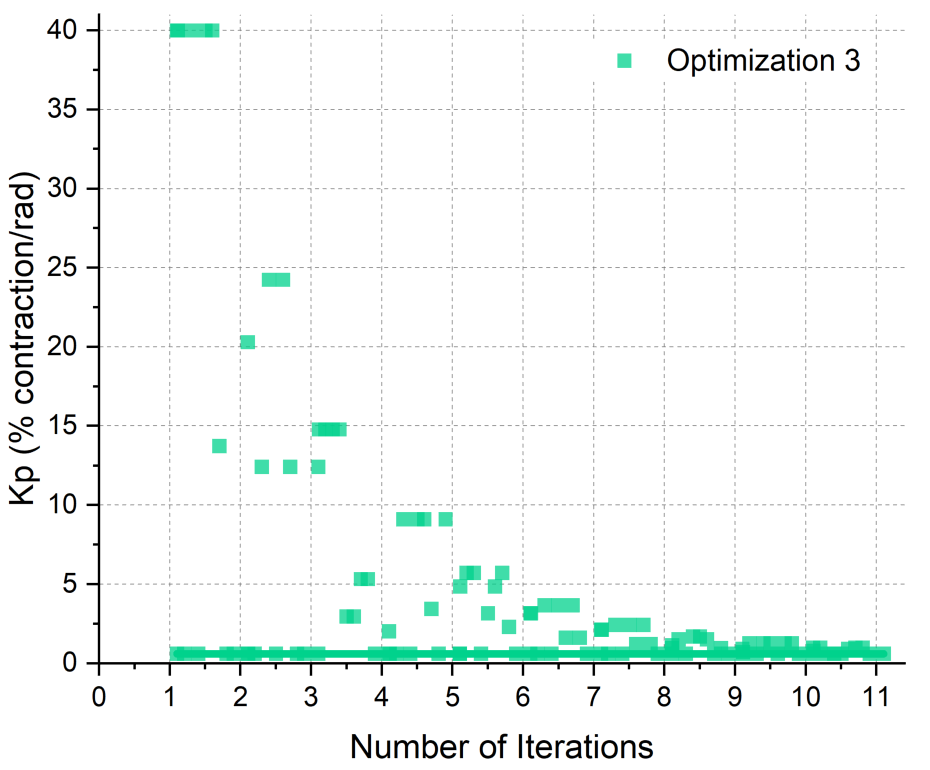

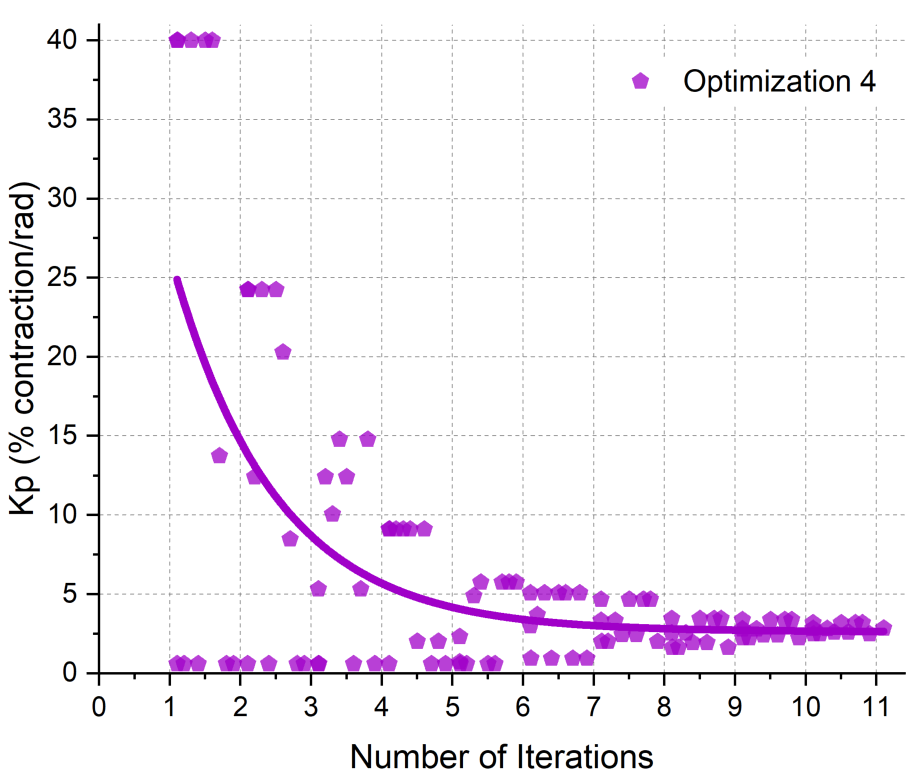


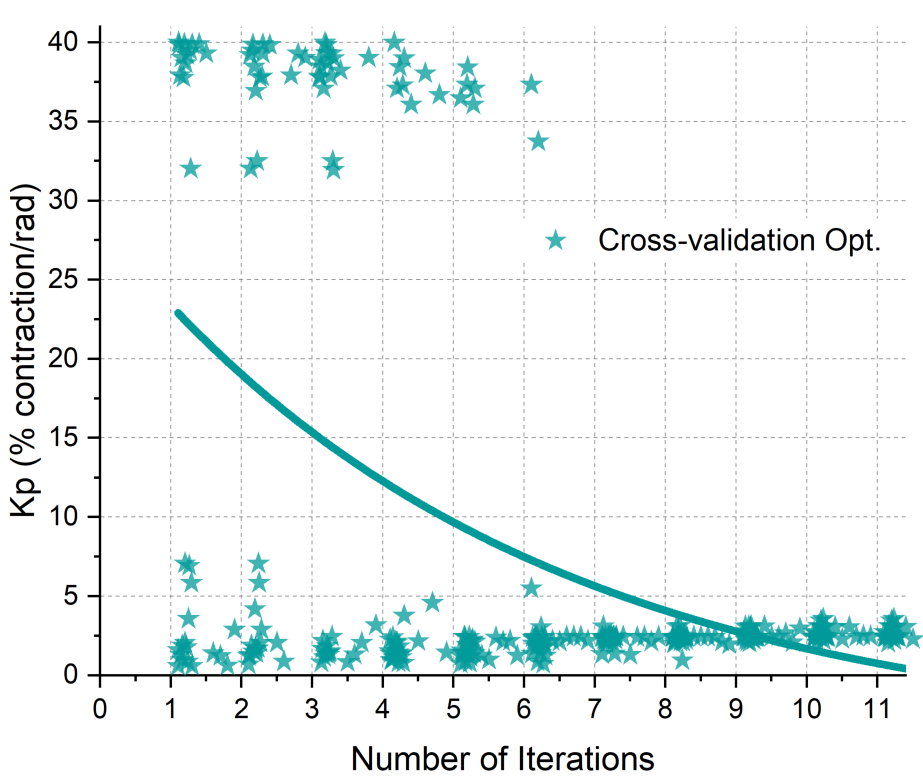


**Convergence Plot of Kd**


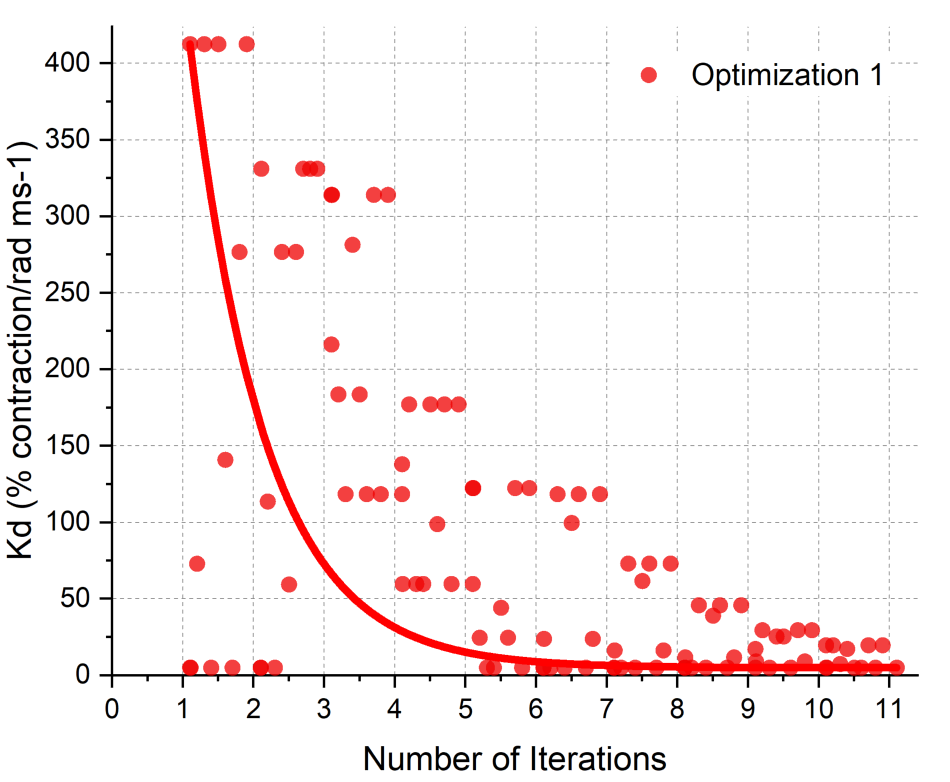

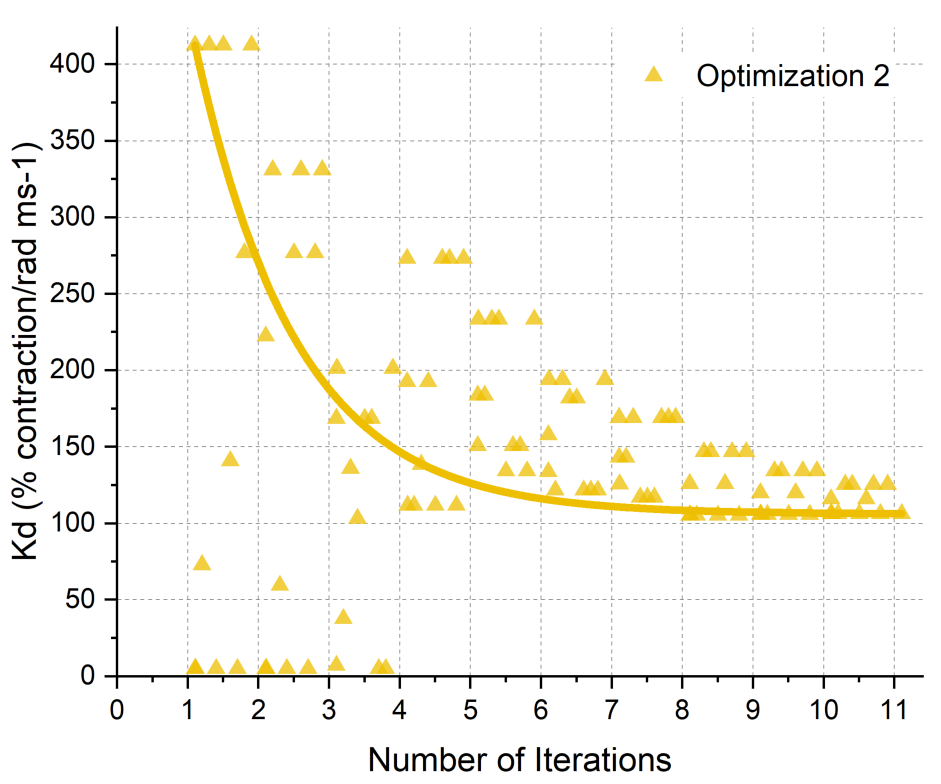


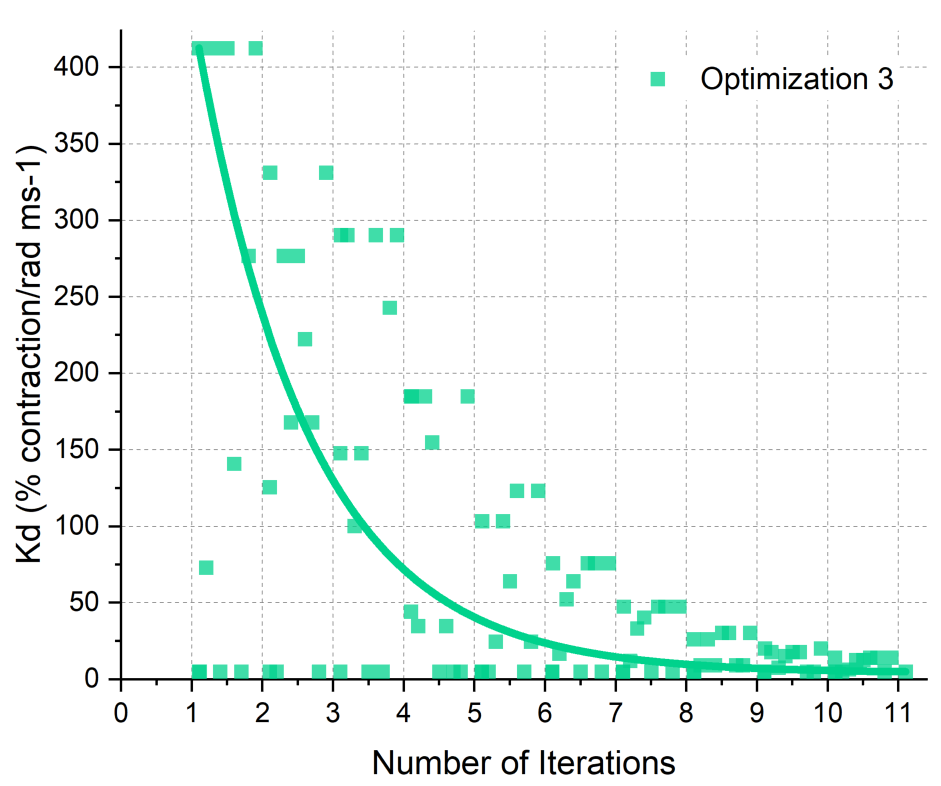

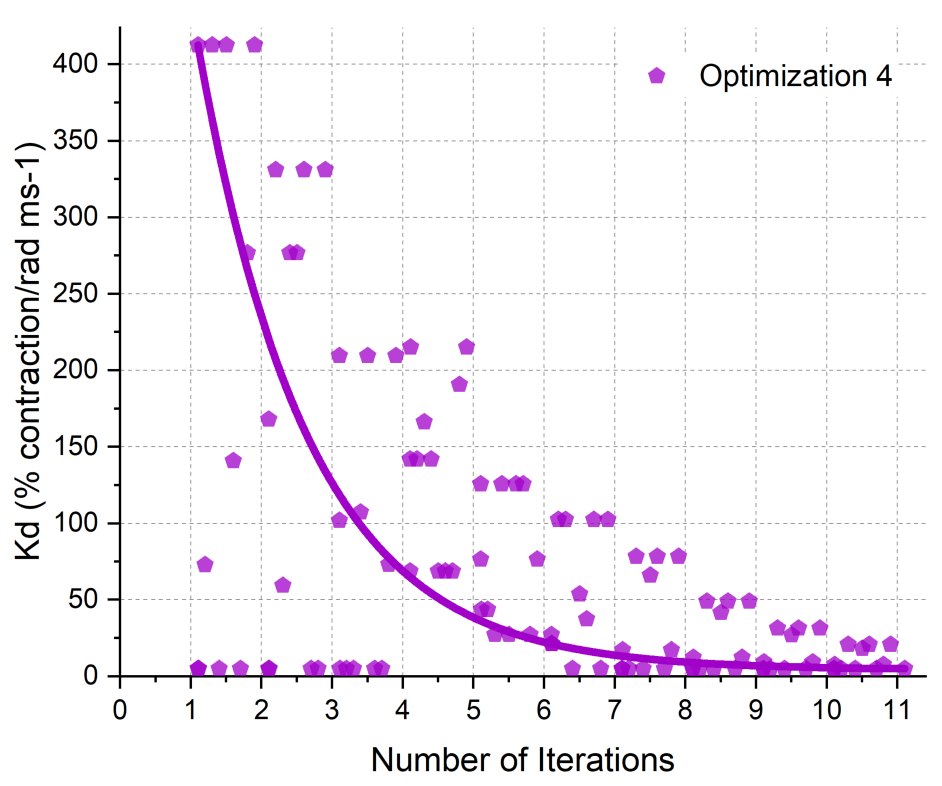


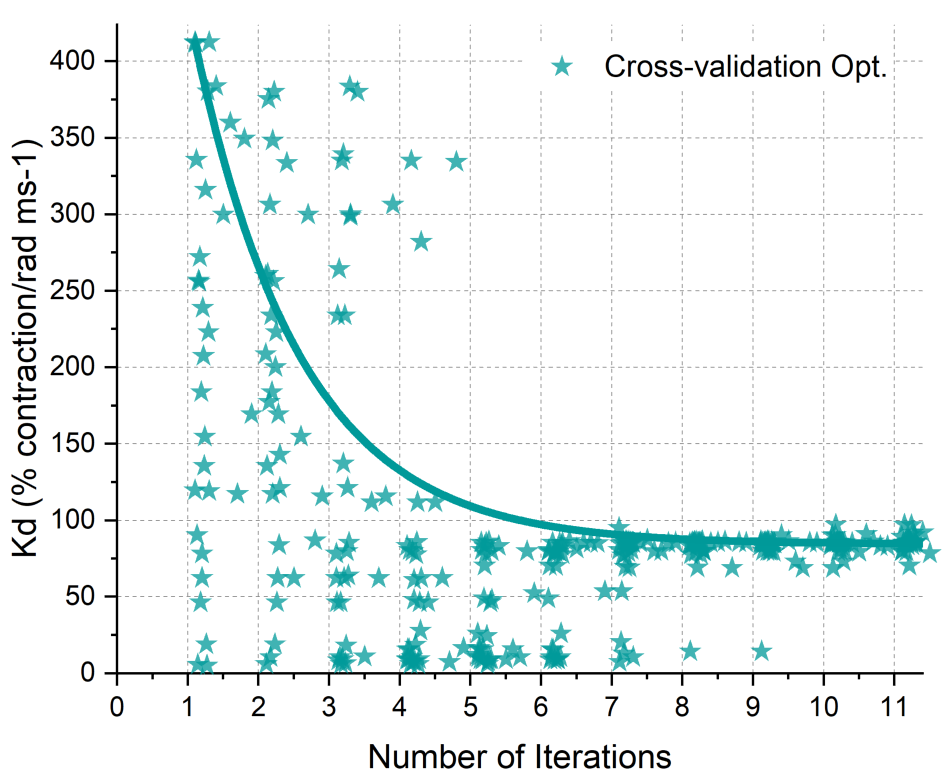


**Convergence Plot of Tnd**


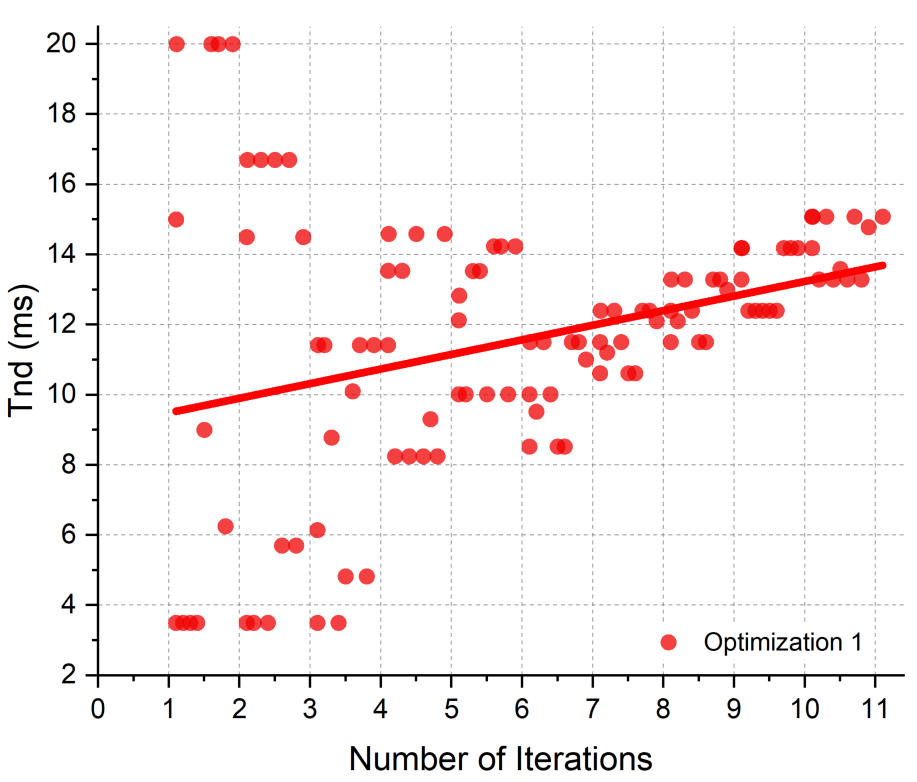

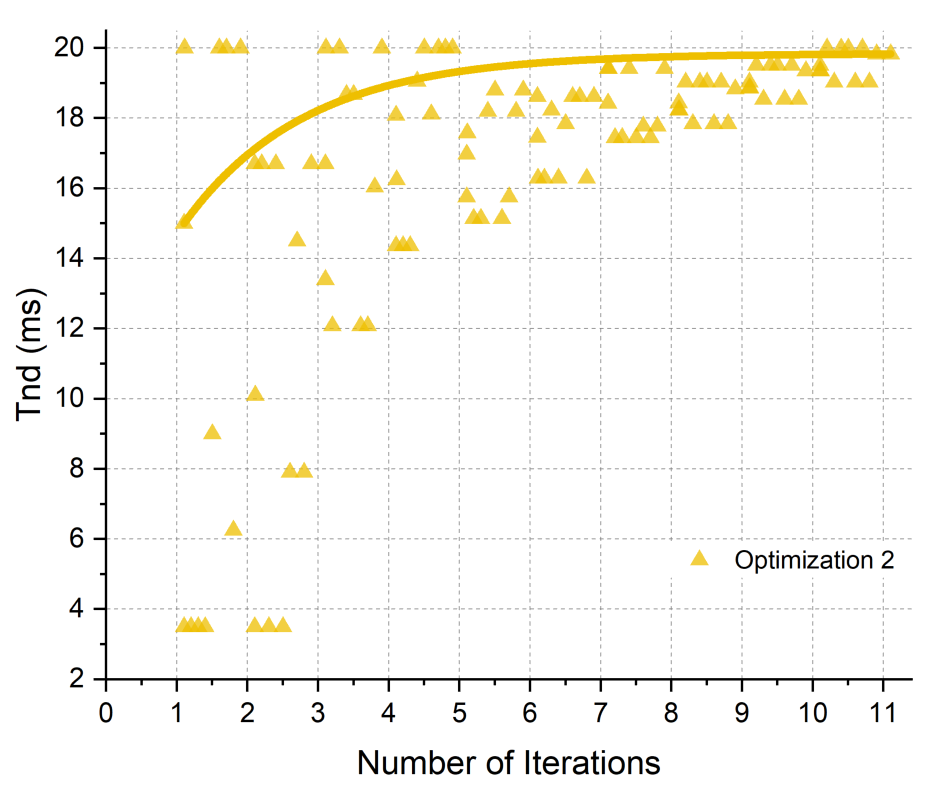


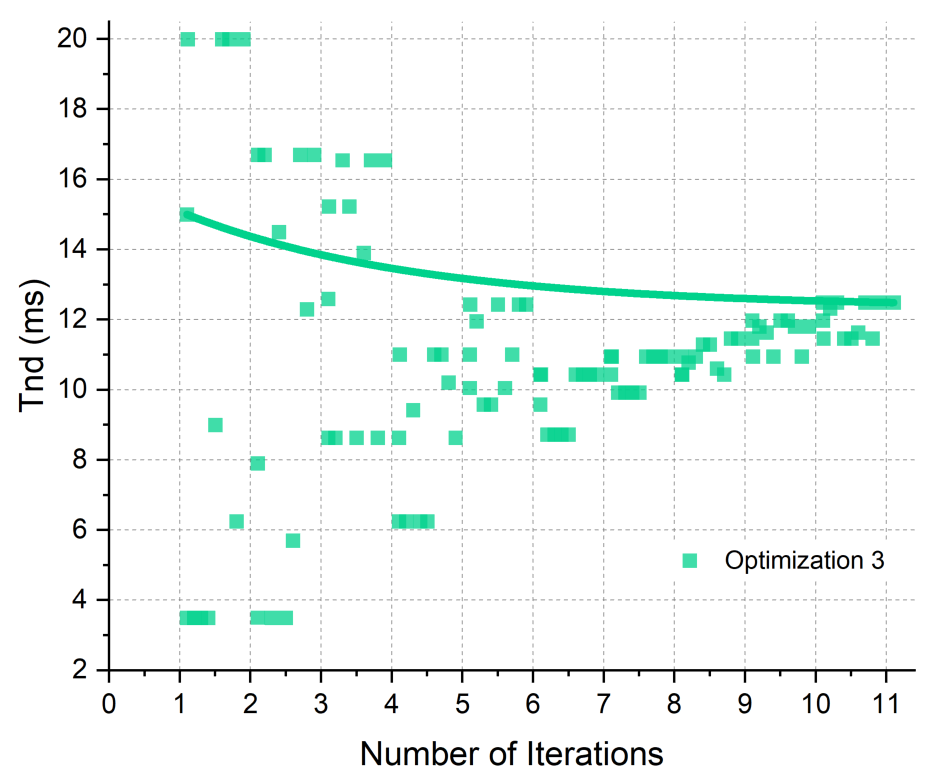

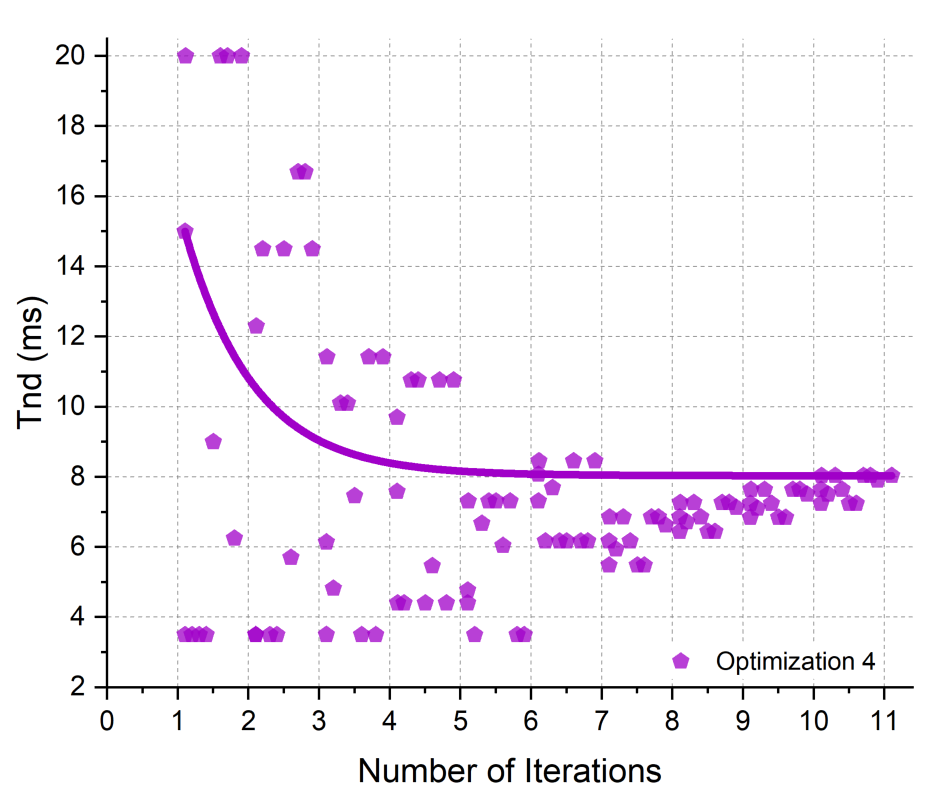


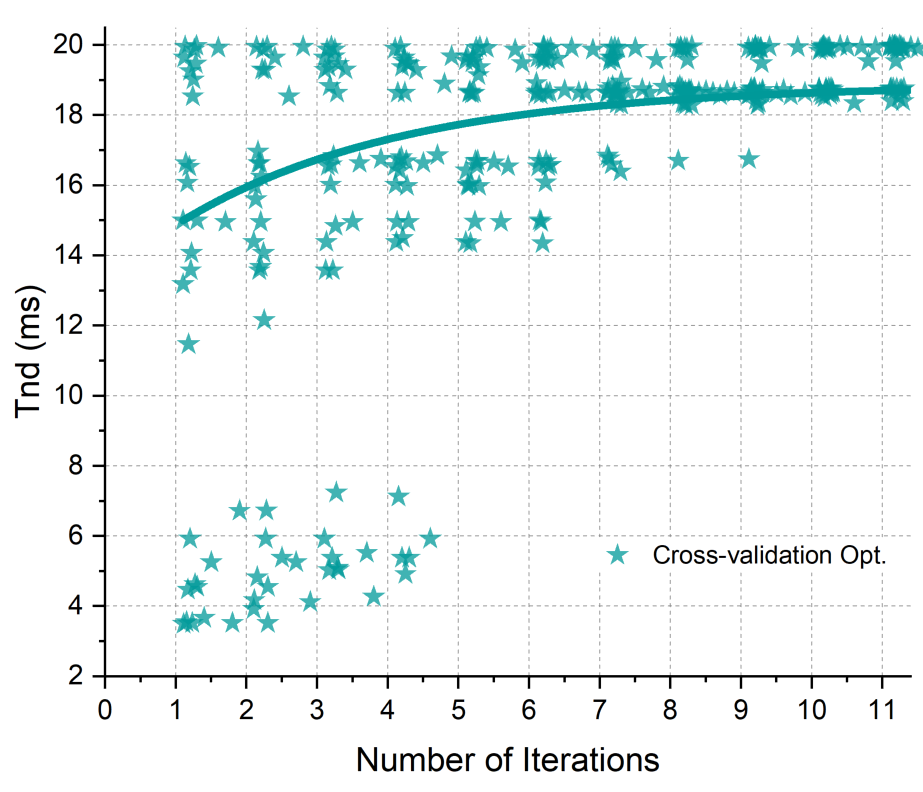


**Convergence Plot of Tna,a**


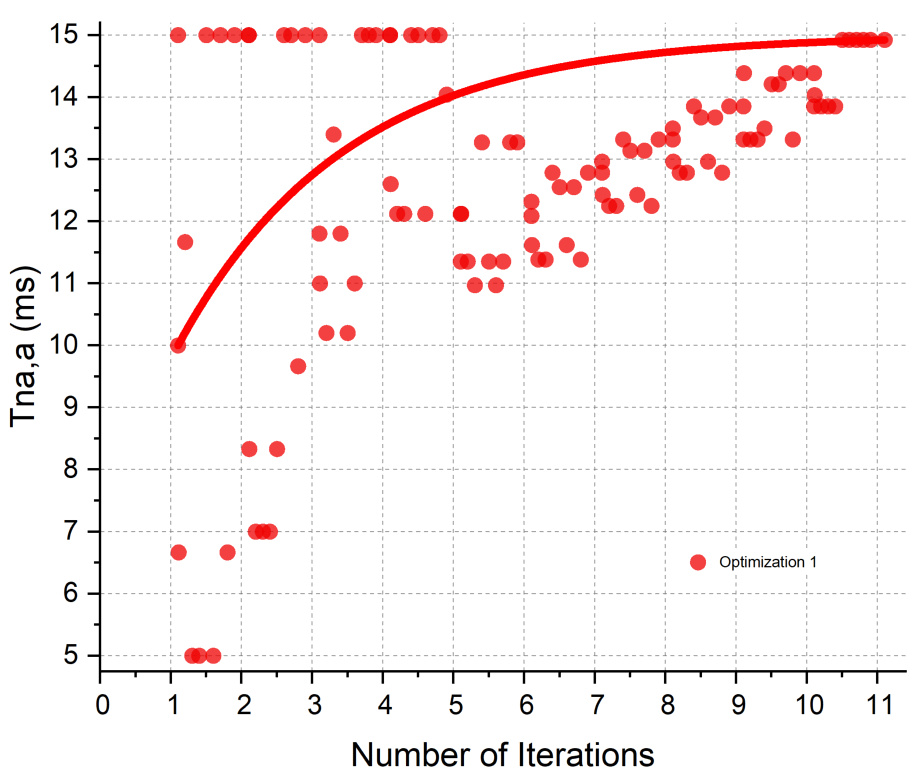

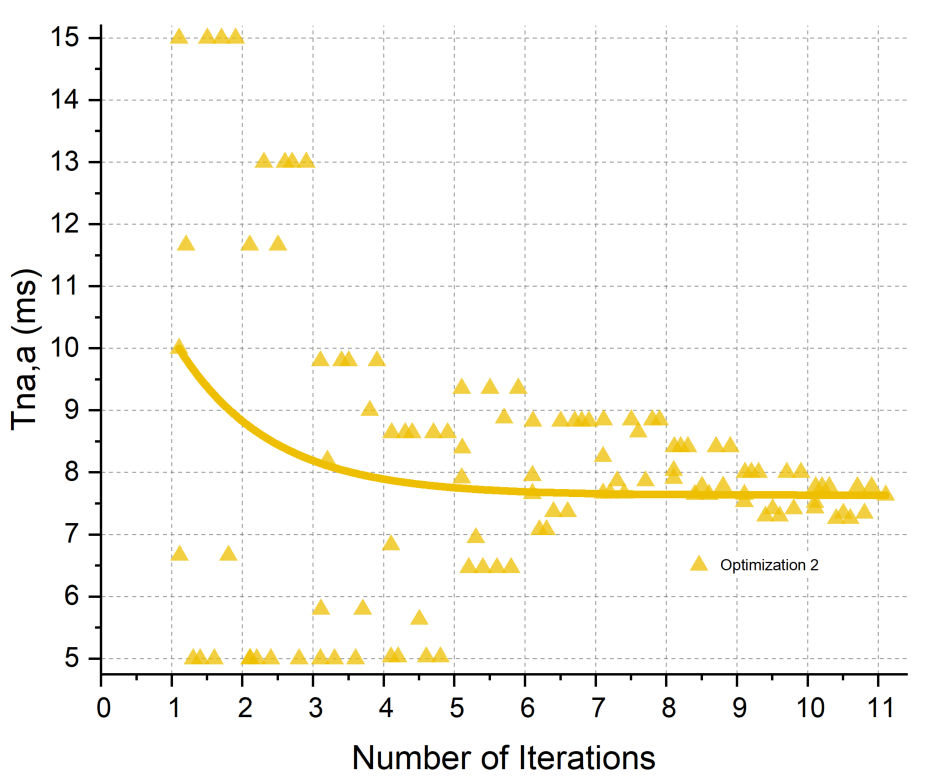


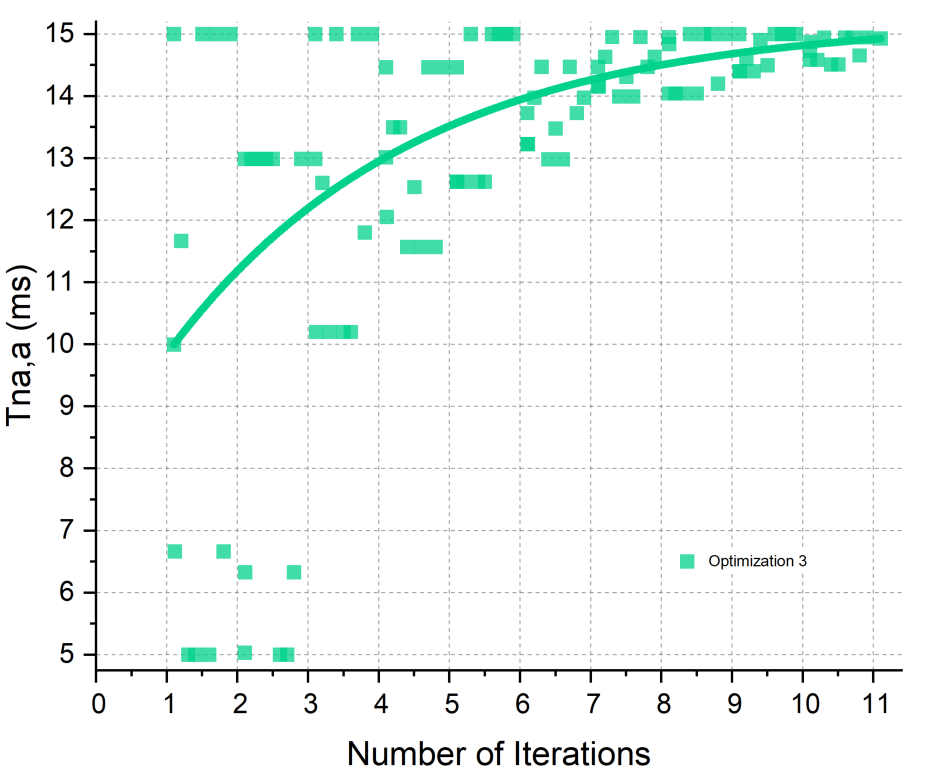

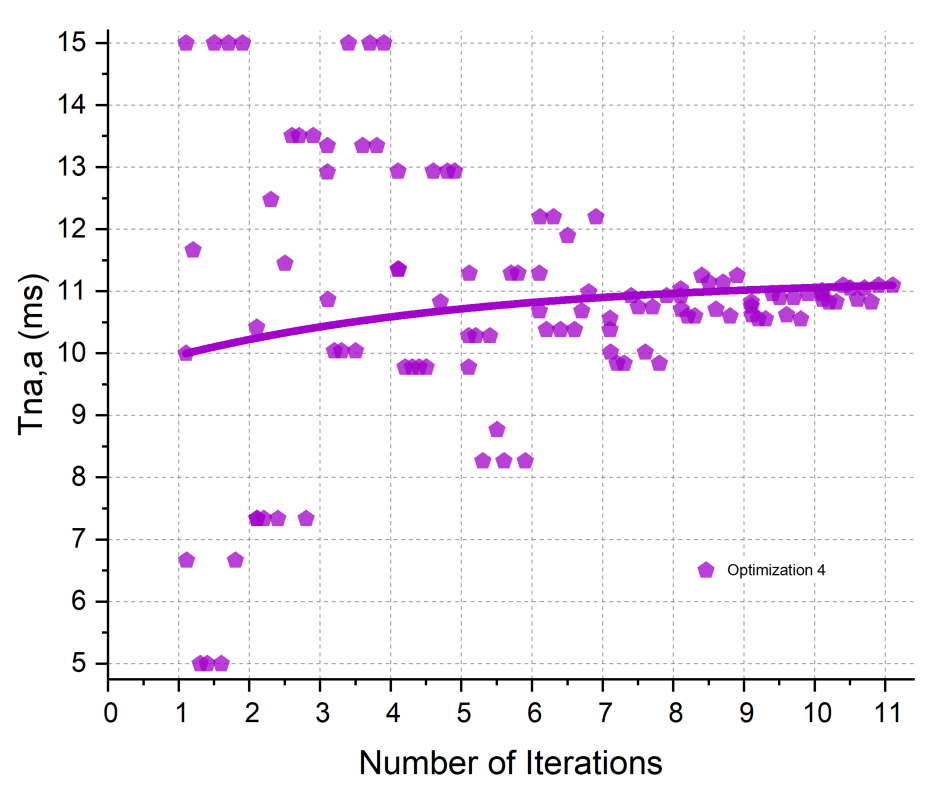


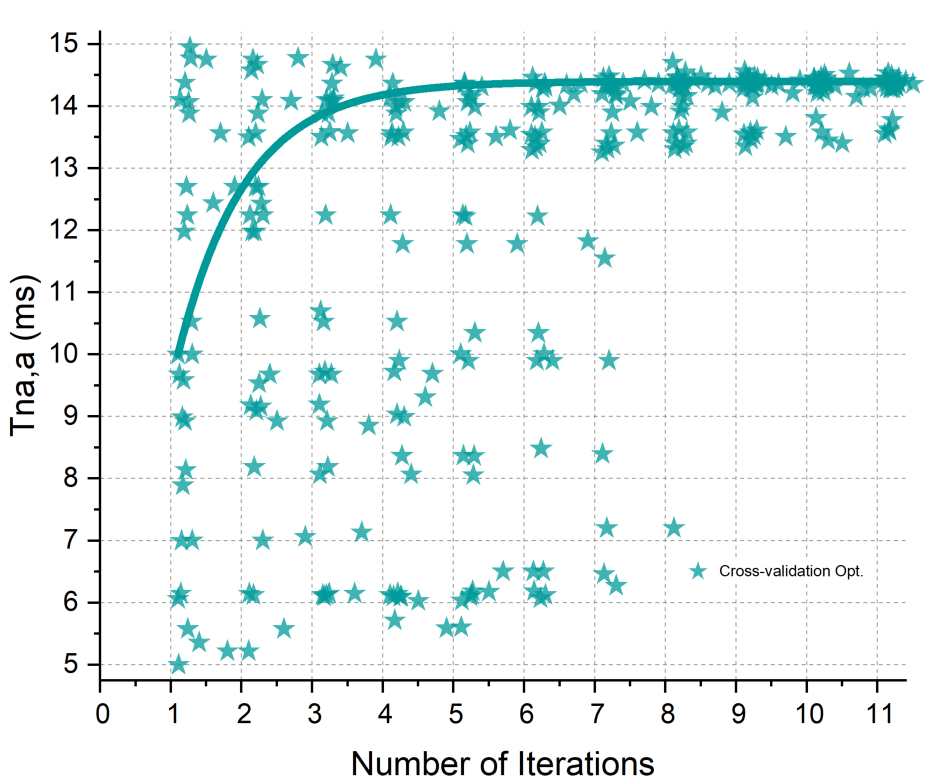


**Convergence Plot of Tna,d**


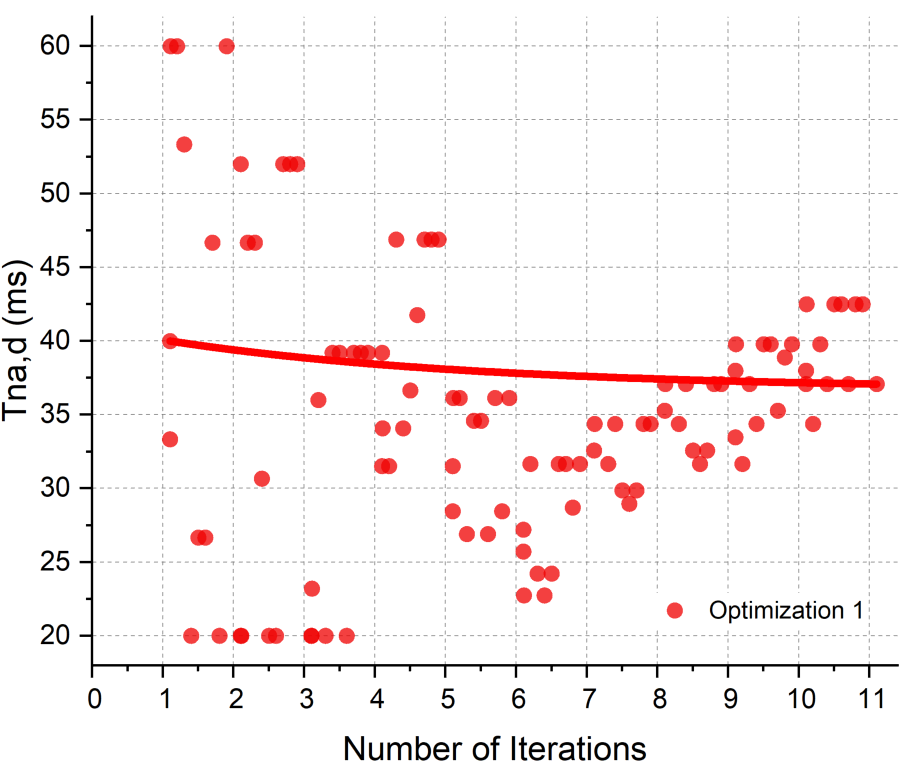

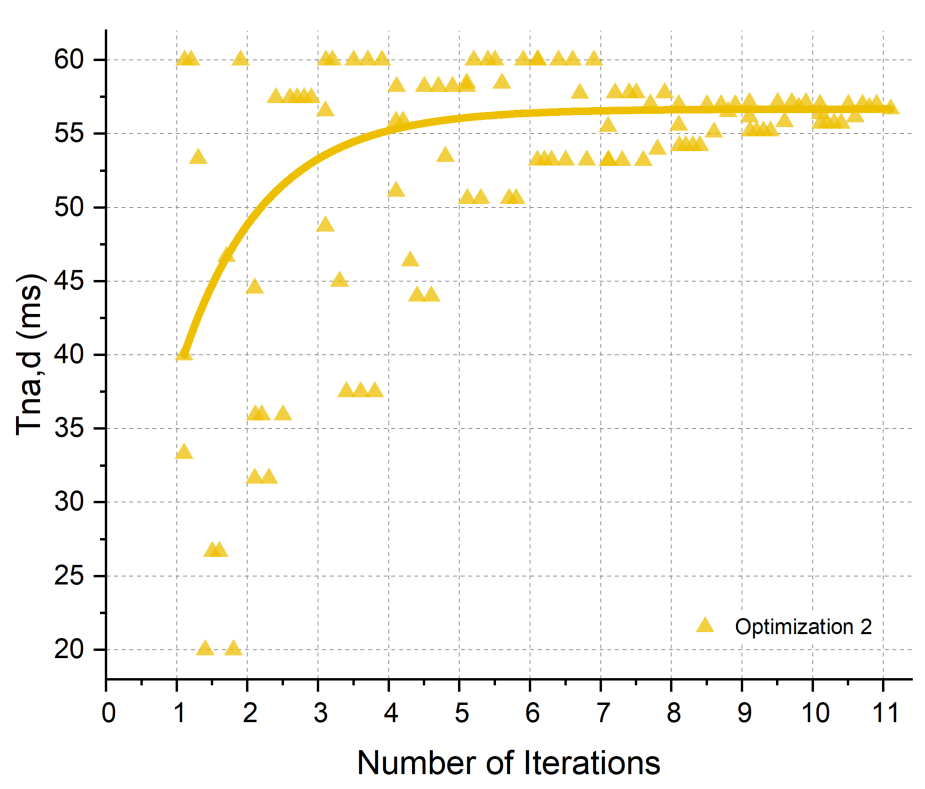


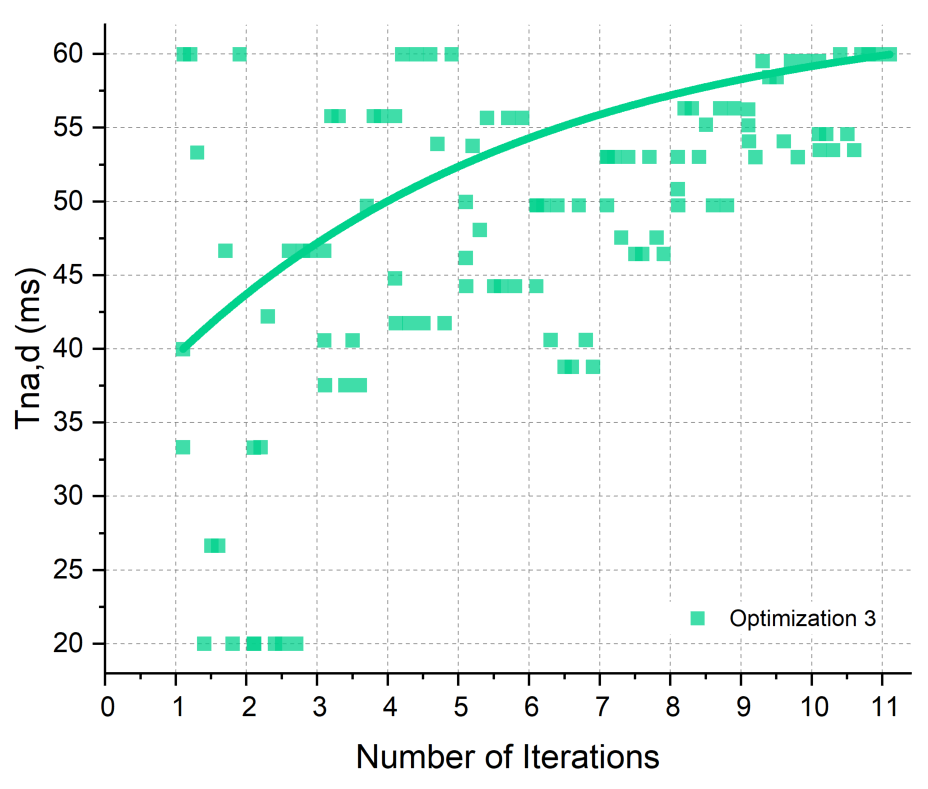

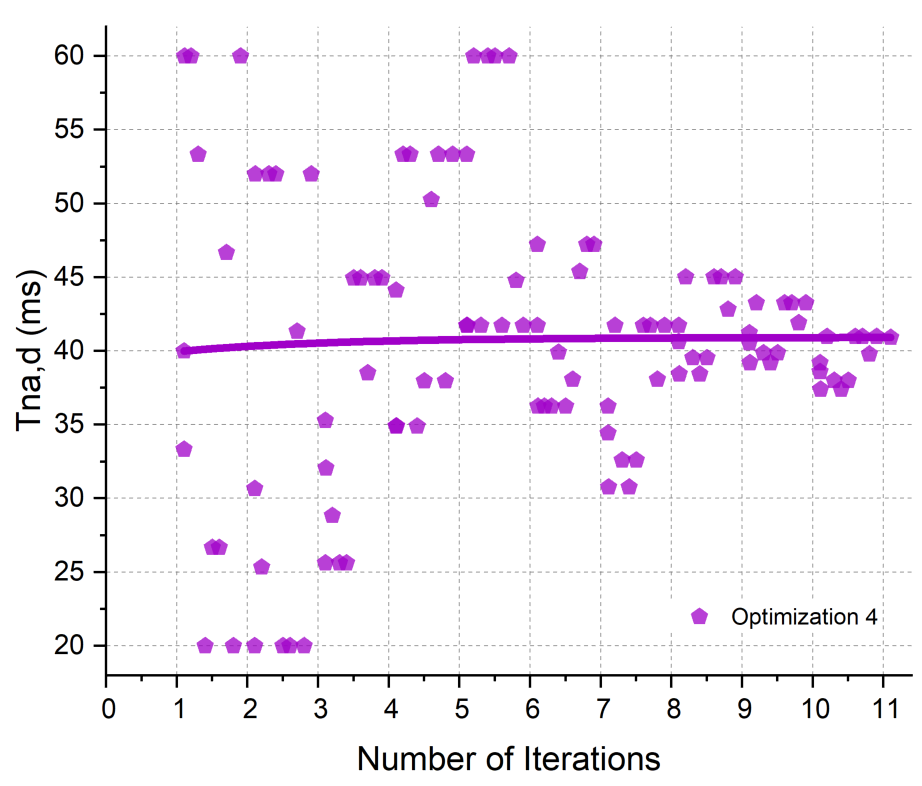


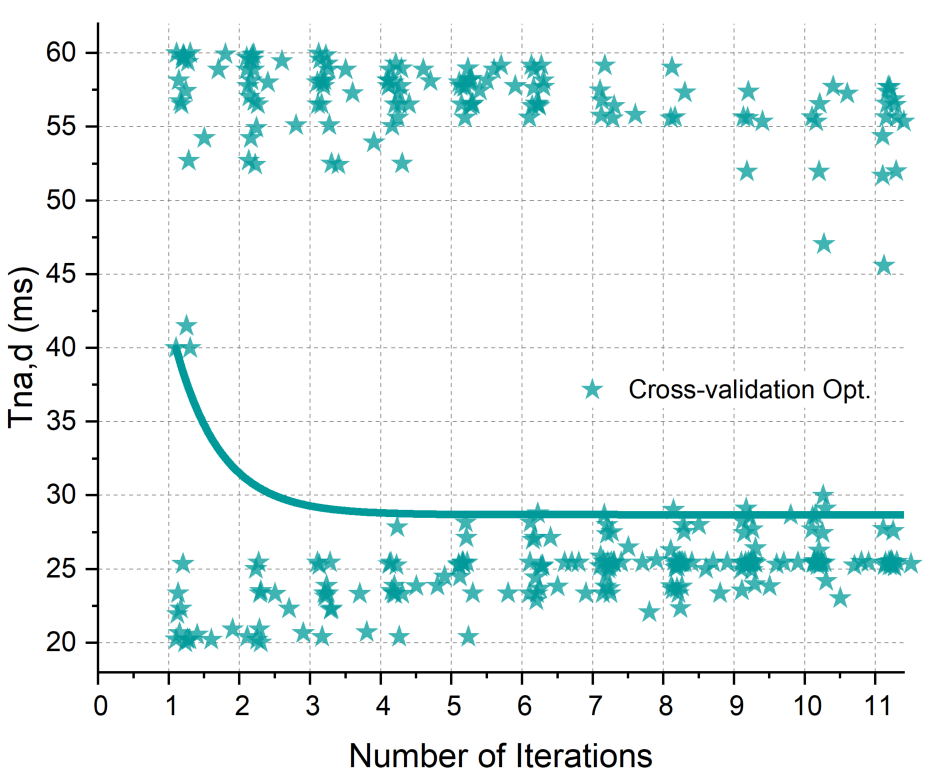


**Convergence Plot of Tne**


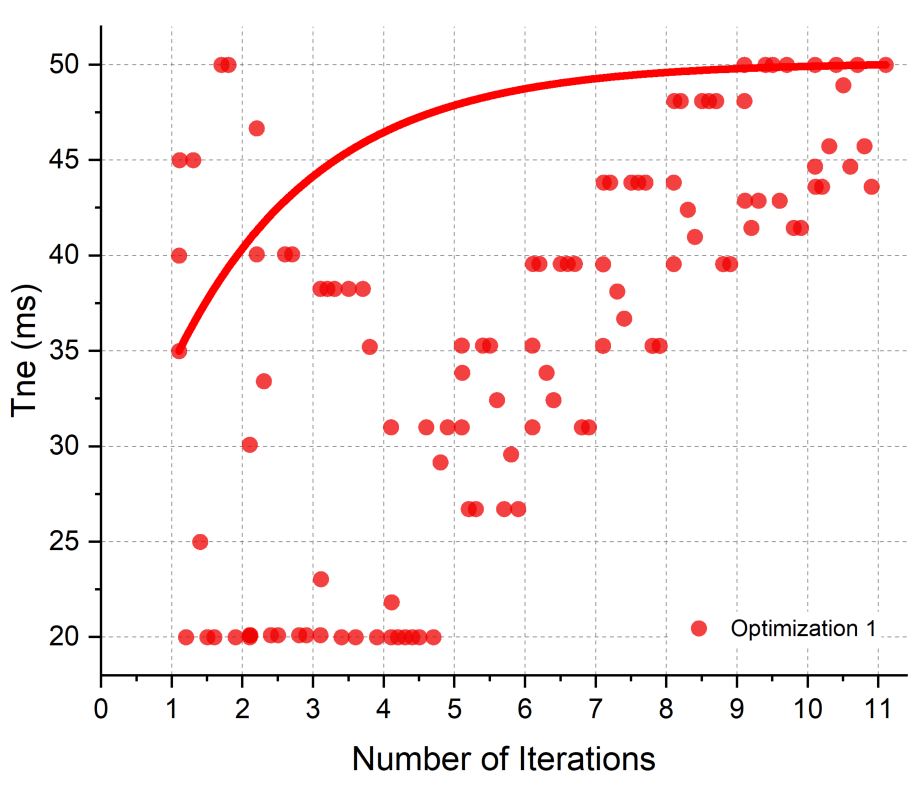

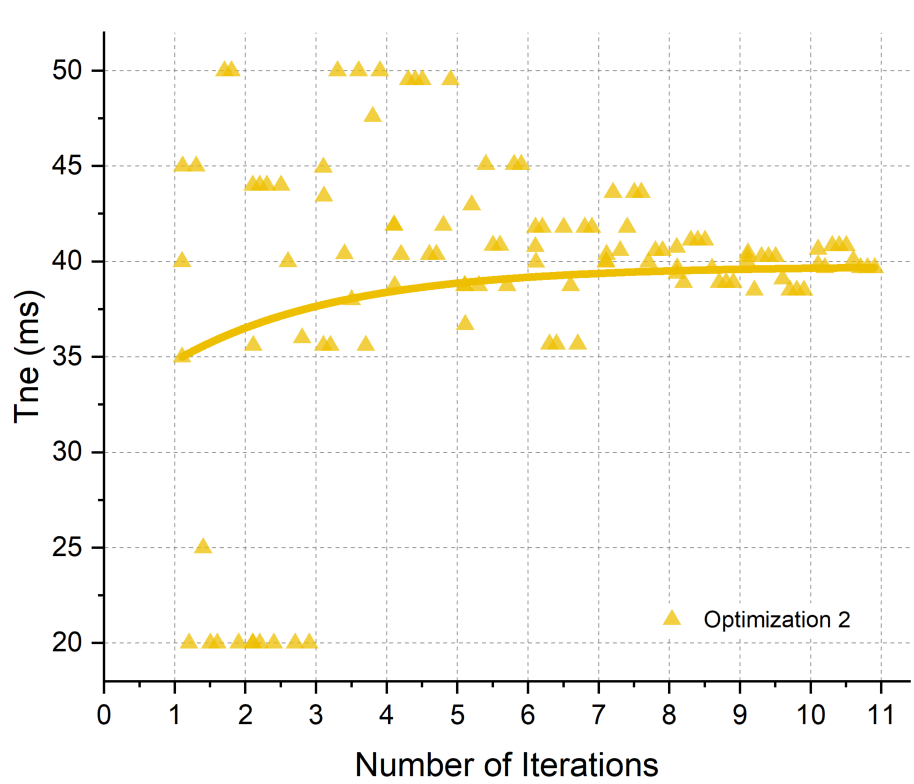


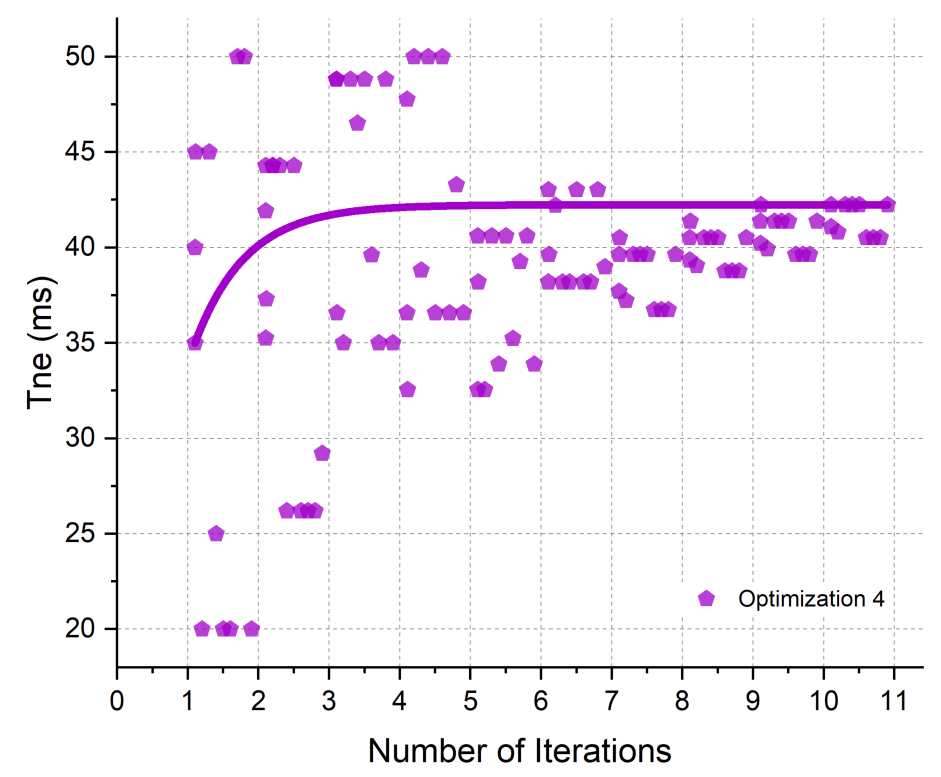

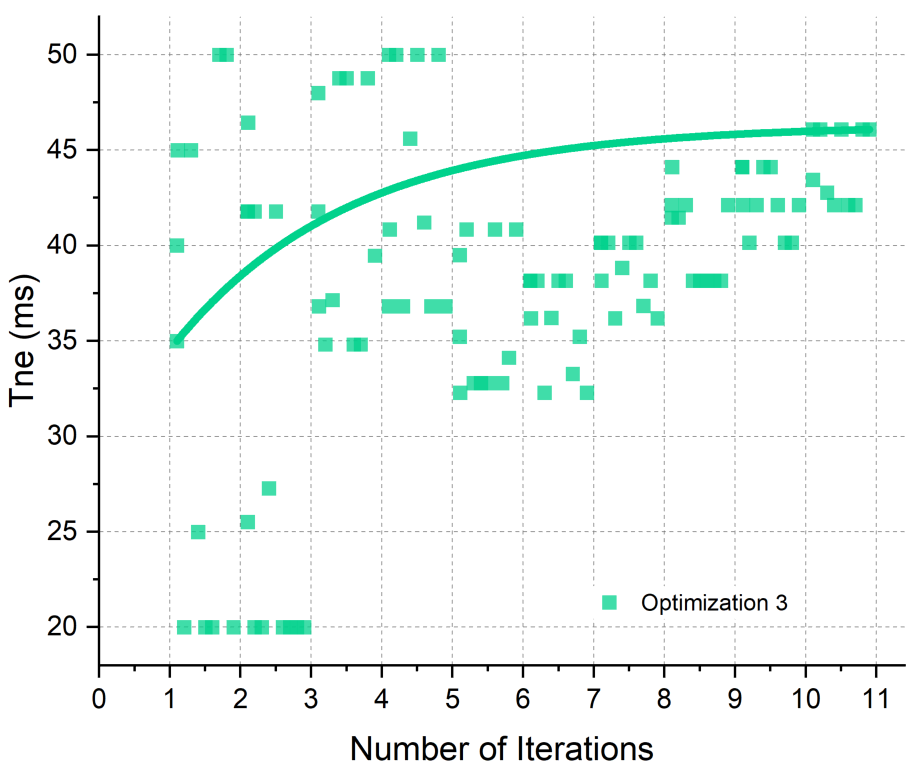


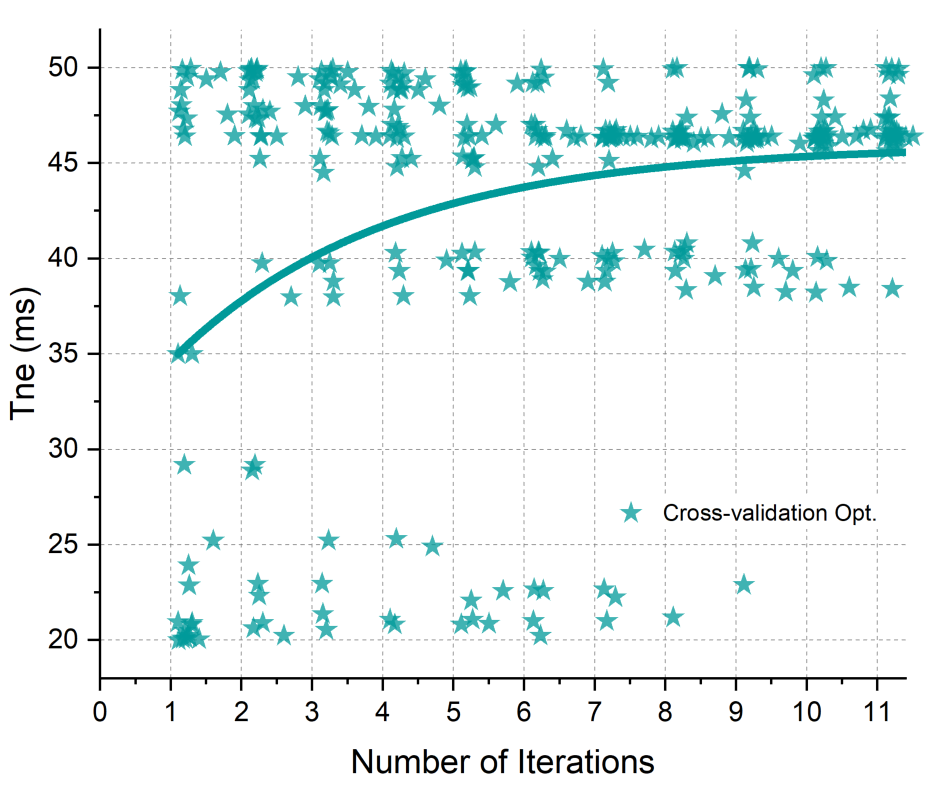

Supplement: Supplementary file 1 — Supplementary material 1 (DOCX 3116 kb) [file 10439_2020_2512_MOESM1_ESM.docx]
